# Spatially resolved transcriptome of the aging mouse brain

**DOI:** 10.1111/acel.14109

**Published:** 2024-02-19

**Authors:** Cheng Wu, Tianxiang Tu, Mingzhe Xie, Yiting Wang, Biao Yan, Yajun Gong, Jiayi Zhang, Xiaolai Zhou, Zhi Xie

**Affiliations:** ^1^ State Key Laboratory of Ophthalmology, Zhongshan Ophthalmic Center Sun Yat‐sen University Guangzhou China; ^2^ State Key Laboratory of Medical Neurobiology, MOE Frontiers Center for Brain Science, MOE Innovative Center for New Drug Development of Immune Inflammatory Diseases Institutes of Brain Science, Institute for Medical and Engineering Innovation, Department of Ophthalmology, Eye & ENT Hospital, Fudan University Shanghai China

**Keywords:** aging, brain, cell types, gene expression, spatial transcriptome

## Abstract

Brain aging is associated with cognitive decline, memory loss and many neurodegenerative disorders. The mammalian brain has distinct structural regions that perform specific functions. However, our understanding in gene expression and cell types within the context of the spatial organization of the mammalian aging brain is limited. Here we generated spatial transcriptomic maps of young and old mouse brains. We identified 27 distinguished brain spatial domains, including layer‐specific subregions that are difficult to dissect individually. We comprehensively characterized spatial‐specific changes in gene expression in the aging brain, particularly for isocortex, the hippocampal formation, brainstem and fiber tracts, and validated some gene expression differences by qPCR and immunohistochemistry. We identified aging‐related genes and pathways that vary in a coordinated manner across spatial regions and parsed the spatial features of aging‐related signals, providing important clues to understand genes with specific functions in different brain regions during aging. Combined with single‐cell transcriptomics data, we characterized the spatial distribution of brain cell types. The proportion of immature neurons decreased in the DG region with aging, indicating that the formation of new neurons is blocked. Finally, we detected changes in information interactions between regions and found specific pathways were deregulated with aging, including classic signaling WNT and layer‐specific signaling COLLAGEN. In summary, we established a spatial molecular atlas of the aging mouse brain (http://sysbio.gzzoc.com/Mouse‐Brain‐Aging/), which provides important resources and novel insights into the molecular mechanism of brain aging.

## Graphical abstract

We generated spatial maps of young and old mouse brains and identified brain spatial domains. We analyzed genes and functional changes in different spatial regions with aging, and revealed changes in cell types in different regions by combining single‐cell data. We detected changes in information interactions between regions. In addition, qPCR and immunohistochemistry were used to verify some genes. This study provides important resources for the mouse brain aging. To facilitate data sharing, we provided an online website for display and data mining.

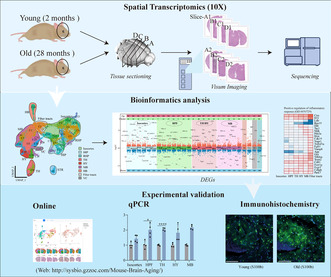

**Abbreviations**
GSEA: gene set enrichment analysis
HB: hindbrain
HIP: hippocampal region
HY: hypothalamus
KEGG: Kyoto Encyclopedia of Genes and Genomes
MB: midbrain
OCT: optimal cutting temperature
PCA: principal component analysis
RHP: retrohippocampal region
ST: spatial transcriptome
TH: thalamus
VC: ventricular systems

## 1 INTRODUCTION

Brain aging is an intricate and irreversible biological process, which is associated with cognitive decline and memory loss and is one of the major risk factors for many neurodegenerative disorders, such as Alzheimer's disease (AD) and Parkinson's disease (PD) (Hou et al., 2019; Kern & Behl, 2009; Lopez‐Otin et al., 2013). The mammalian brain is divided into distinct structural regions that perform diverse functions (Genon et al., 2018; Ortiz et al., 2020; Power et al., 2011). Due to functional differences, brain regions show differential responsiveness to aging across the lifespan (Berchtold et al., 2008; Luo et al., 2020).

Emerging evidence from human and animal models suggests that aging processes rely on precise spatiotemporal regulation of gene expression in brain (Berchtold et al., 2008; Fraser et al., 2005; Weindruch & Prolla, 2002). Multiple studies have reported that neuron‐specific genes are predominantly downregulated in all brain regions with aging, and these genes display region‐specific, age‐dependent expression changes by preserving their regional identity (Erraji‐Benchekroun et al., 2005; Somel et al., 2010; Tollervey et al., 2011). Moreover, increasing evidence suggests that brain aging is regulated by the interaction of multiple brain regions, which must work together to control complex physiological processes (Berchtold et al., 2013). These studies provided important clues into the relationship between gene, region and function.

Spatial transcriptome (ST) is a recently developed molecular profiling method that measures gene activity with location information in a tissue sample (Stahl et al., 2016). Using ST, Ortiz et al. (2020) established a molecular atlas to define the spatial organization of adult mouse brain regions. Navarro et al. (2020) revealed genes associated with dysregulated mitochondrial functions and stress signaling in Alzheimer's disease (AD). Kiss et al. (2022) revealed inflammatory foci defined by senescent cells in the white matter, hippocampi, and cortical gray matter in the aged mouse brain. Despite these studies have conducted preliminary exploration of brain functions to structures at the molecular level, a full spatial transcriptome in aging brain is unclear. Changes in gene expression and cell type in specific spatial regions in aging brain have not been systemically explored.

In this study, we generated a ST atlas for young and old mouse brain. We identified 27 clear molecular domains in the mouse brain. We characterized and validated spatial‐specific changes in gene expression and pathways with aging. Combined with single‐cell transcriptome in the mouse brain, we further characterized the spatial‐specific changes in cell types. Finally, we deciphered ligand‐receptor interactions involving pathways among the brain regions during aging. Our analysis provided many novel biological insights into brain aging. Overall, we established a brain aging spatial molecular atlas and provided many novel biological insights into the complex aging process. Our datasets and analysis are accessible at http://sysbio.gzzoc.com/Mouse‐Brain‐Aging/.

## 2 MATERIALS AND METHODS

### 2.1 Brain sections of young and old mice

Two young (2 months old) and two old (28 months old) mice (strain C57BL/6J) were euthanized with an overdose of isoflurane. The brains were rapidly extracted from the cranial cavity, and immediately submerged in ice‐cold artificial cerebrospinal fluid. The brains were then blotted to remove excess liquid and were subsequently embedded in optimal cutting temperature (OCT) compound (SAKURA). The brains with OCT compound were quick‐frozen on dry ice immediately and stored at −80°C until cryosectioning. The cryosectioning was performed in a cryostat (Leica, CM1950) to cryosect the OCT embedded tissue blocks into appropriately sized sections for Visium Spatial slides while keeping the samples frozen. Brain sections were each 10 μm thick. The Institutional Animal Care and Use Committee of Fudan University approved the experiments.

### 2.2 Fixation, staining, and imaging

We selected four slices in each group, which were located near 63S, 85S, 88S, and 95S of ABA. The four slices cover the key regions of the mouse brain, including the cerebral cortex, hippocampus, thalamus, midbrain, hindbrain and fiber tracts. To accurately determine the position of slices, we performed H&E staining. Brain research experts compared the anatomical structural features (such as ventricles, corpus callosum, hippocampus) and cell density with coronal sections of mouse brains from ABA, and then selected appropriate adjacent slices for spatial transcription experiments. The sections were placed within the frames of capture areas on Visium Spatial slides (10X Genomics). Brain section slides were incubated for 1 min at 37°C and then fixed in methanol at −20°C for 30 min. For staining, the slides were incubated in hematoxylin for 7 min and in Bluing Buffer for 2 min. Then, eosin was added to the slides and incubated for 1 min. After each staining step, the slides were washed with DNase‐ and RNase‐free water. Stained brain sections were imaged by a microscope (ECLIPSE Ti, Nikon).

### 2.3 Tissue prepermeabilization

Prepermeabilization was performed to optimize the suitable permeabilization time. Visium Spatial Tissue Optimization Slides & Reagent Kits (10X Genomics) were used for prepermeabilization. The brains were permeabilized in Permeabilization Enzyme for varying amounts of time, and Fluorescent RT Master Mix was added to the brain sections. The tissue sections were incubated in Tissue Removal Mix for 60 min at 56°C. The best permeabilization time (12 min) was selected using a fluorescence microscope (ECLIPSE Ti, Nikon).

### 2.4 Permeabilization and spatial transcriptomic sequencing

Permeabilization and spatial transcriptomics sequencing were performed using Visium Spatial Gene Expression Slides & Reagent Kits. In short, the stained slides were incubated in RT Master Mix for 45 min at 53°C for reverse transcription after permeabilization for the appropriate time (12 min). Next, Second Strand Mix was added to the tissue sections on the slide and incubated for 15 min at 65°C to initiate second strand synthesis. After transfer of cDNA from the slices, barcoded cDNA was purified and amplified. The amplified barcoded cDNA was fragmented, A‐tailed, ligated with adaptors and index PCR amplified. The final libraries were quantified using the Qubit High Sensitivity DNA assay (Thermo Fisher Scientific), and the size distribution of the libraries was determined using a High Sensitivity DNA chip on a Bioanalyzer 2200 (Agilent). All libraries were sequenced by Illumina NovaSeq6000 on a 150‐bp paired‐end run.

### 2.5 10X genomics space ranger pipeline

Illumina BCL files were converted to fastq files by using the 10X Genomics Space Ranger pipeline spaceranger mkfastq function (https://support.10xgenomics.com/). Next, fastq files were aligned to the 10 mm reference and manually aligned to the respective H&E‐stained images using spaceranger count (spaceranger v 1.2.0) to obtain the spatial transcriptional profiles. The number of spots, average reads and median genes in each section are listed (Table S1).

### 2.6 Single‐cell spatial transcriptomics data processing

The gene expression matrix, image of the tissue slice and scaling factors were loaded into Seurat package (v3.2.0; https://github.com/satijalab/seurat) using the “Load10X_Spatial” function. We first normalized the slice data using “SCTransform” function to regress out the variance in the sequencing depth across data points and then merged the data from eight slice objects using “Merge” function. The merged dataset was reduced into a lower dimensional space using principal component analysis (PCA). The top 30 principal components (PCs) were used as inputs for graph‐based cell clustering at a resolution value of 0.8 using the “FindNeighbors” and “FindClusters” functions. The clustering results were visualized using Uniform Manifold Approximation and Projection (UMAP) with Seurat functions RunUMAP.

### 2.7 Allen brain atlas (ABA)

**FIGURE 1:**
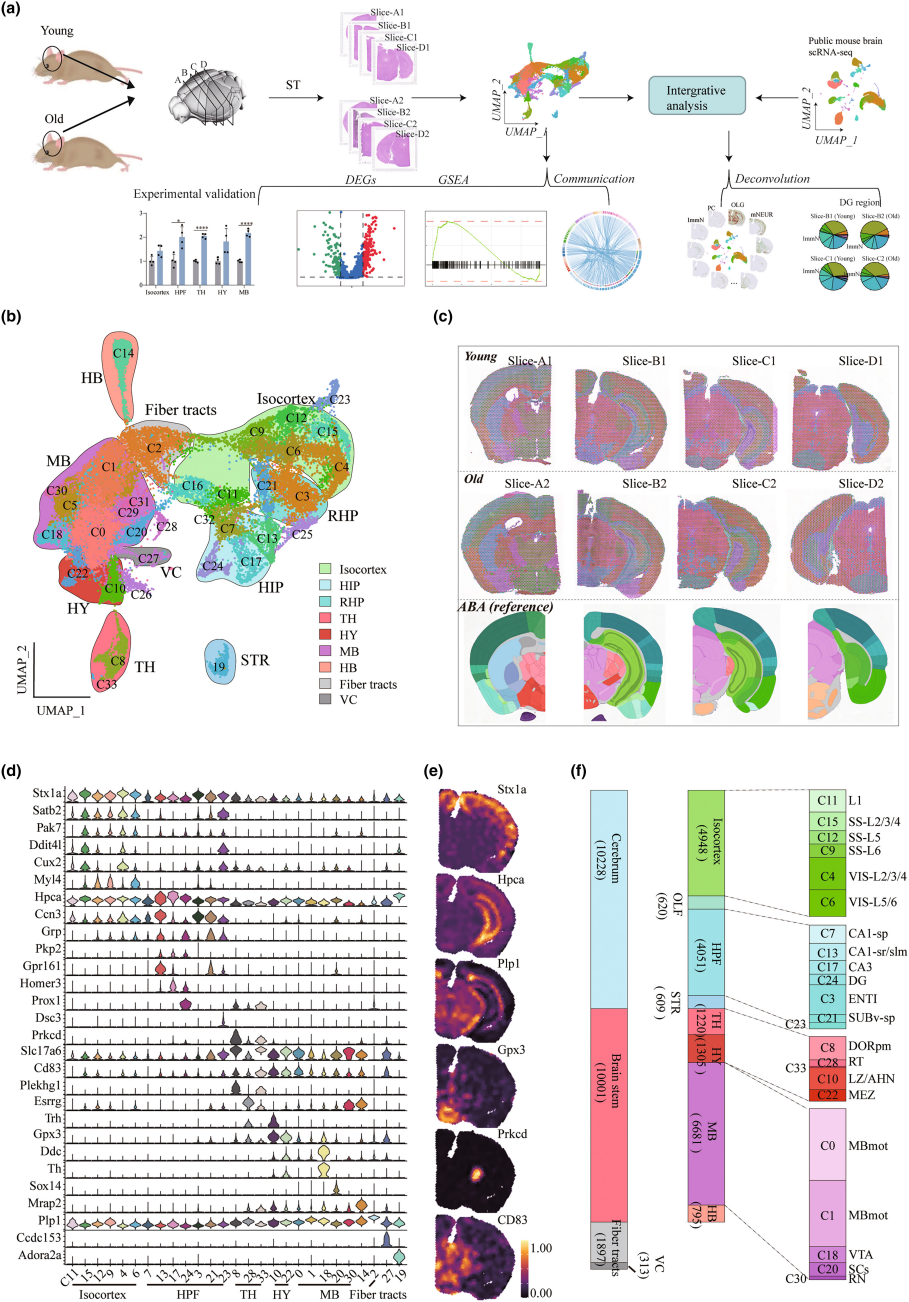
Spatial transcriptional mapping of young and old mouse brains. (a) Overview of the workflow. We performed spatial transcriptome (ST) sequencing on a total of 8 slices of young and aged mouse brains, and then used bioinformatics analysis methods to identify spatial domain and aging‐related genes. (b) Uniform manifold approximation and projection (UMAP) visualization of 27,089 spots, colored by clusters. Hippocampal region (HIP); Retrohippocampal region (RHP); Thalamus (TH); Hypothalamus (HY); Midbrain (MB); Hindbrain (HB); Ventricular systems (VC). (c) Visualization of spot clusters on 8 spatial slices; ABA divides regions based on anatomy (as a reference) (bottom). (d) Violin diagram of the expression of known brain region‐related genes in spot clusters. Hippocampal formation (HPF, including HIP and RHP). (e) The expression distribution of region‐related genes on slice‐B1. (f) Distribution of spots according to region definitions.

The Allen Institute for Brain Science established a complete brain atlas with information on tissue coordinates, the reference ABA neuroanatomical definitions, and spatially defined gene expression patterns (www.brain‐map.org). We obtained four sets of coronal slices (spatial transcriptome sequencing) that approximately corresponded to slices on the Allen Mouse Brain Reference Atlas (ABA) through comparison (Figure 1c). In addition, we obtained the expression distribution of some region‐related marker genes on ABA slices through a gene search.

### 2.8 Differential expression analysis

After initial quality control preprocessing and determination of cellular identities, we used the “FindMarker” function from the Seurat package to perform DGE analysis with test.use = “wilcox”. Here, we set two sets of parameters when calculating the significance of differential expression. When identifying the differentially expressed genes between different regions, we set the default threshold (logFC = 0.25). When calculating the differentially expressed genes in the same region with aging, we set a threshold (FC ≥10%, *p* < 0.05), considering that the gene expression changes were relatively small.

### 2.9 Identification and naming rules of brain regions

To define and name the molecular regions of the brain, we used two dimensions of information. Based on the cluster analysis of the single‐cell spatial transcriptional profile, we divided the spots into different molecular clusters (33 clusters). The potential marker genes contained in the 33 clusters were obtained through differential expression analysis. Therefore, we named the clusters considering the expression of marker genes and the main position of the spots in the ABA anatomical region. We first divided the clusters into large areas, including the isocortex, olfactory areas (OLF), hippocampal formation (HPF), interbrain (IB), midbrain (MB), hindbrain (HB), fiber tracts, and ventricular systems (VS). If the cluster contained spots in a single anatomical region and the marker genes are also known to be highly expressed genes in this area, we directly used the anatomical area name. If the cluster contained spots covering multiple anatomical regions and the marker gene expression was significant, the major region was used. If the cluster contained the scattered spots that covered the entire slice, we defined it as other.

### 2.10 KEGG and GO

Kyoto Encyclopedia of Genes and Genomes (KEGG) pathway changes in each region during aging by performing gene set enrichment analysis (GSEA) (Subramanian et al., 2005). Briefly, we used “Seurat::FindMarkers” function with parameters (min.pct = 0.25, logfc.threshold = 0) to calculate gene expression differences (old vs. young) and sorted genes, and then used “msigdbr::msigdbr” function to define gene sets with parameters (species = “Mus musculus”, category = “C2”), selecting KEGG gene dataset as a reference. Finally, we used “fgsea::fgsea” function to calculate the *p* values for each pathway with nperm = 1000. Only gene sets with *p* < 0.05 were considered as significantly enriched. GO enrichment analysis used the clusterProfiler R package (Yu et al., 2012). GO:0090398 related genes can be downloaded from http://www.informatics.jax.org/vocab/gene_ontology/GO:0090398, and GO:0050729 related genes can be downloaded from http://www.informatics.jax.org/vocab/gene_ontology/GO:0050729.

### 2.11 Public dataset of single‐cell sequencing of aging mice brains

We downloaded the raw single‐cell RNA sequencing datasets from NCBI's Gene Expression Omnibus (GEO) under the accession number GSE129788. The datasets were from 16 mouse brains (8 young and 8 old) and contained 37,089 processed single cells. Young male mice were used at 2–3 months, and old male mice were used at 21–22 months of age. They performed single‐cell sequencing of the whole brain without distinguishing regions. To be consistent with the spatial transcriptome data processing method, we denormalized the normalization matrix and used SCT to renormalize. Next, PCA was carried out with default parameters. Clustering was performed with the clustering resolution set to 0.6, and 27 cell clusters were identified. Using the marker genes of known cell types provided in the original finding (Ximerakis et al., 2019), we defined these 27 clusters and found that the cell types were similar to the original text, except for identifying several subtypes (Figure S5).

### 2.12 Deconvolution of the spatial transcriptome

To integrate the data of single cell and spatial transcriptome data, deconvolution was performed by using SPOTlight (Elosua‐Bayes et al., 2021), which is a nonnegative matrix factorization (NMF)‐based spatial deconvolution framework. SPOTlight identifies signature genes from scRNA‐seq data based on the major cell type annotation by using Seurat::FindAllMarkers (assay = “SCT”, logfc.threshold = 1, min.pct = 0.9), which are subsequently used to deconvolute ST spots by using spotlight_deconvolution (se_sc = SC, counts_spatial = ST, cluster_markers = signature_genes, clust_vr = “Celltype”, cl_*n* = 100, hvg = 3000, transf = “uv”, method = “nsNMF”, min_cont = 0.09). After calculating the cell composition ratio of each spot, the ratio was added to the spatial meta information, and Seurat::SpatialFeaturePlot was used to visualize the spatial distribution of the cell types.

### 2.13 Information interaction based on spatial transcriptome

CellChat (Jin et al., 2021) (V1.1.2) was used to infer the cell–cell communication and significant pathways by integrating gene expression with prior knowledge of the interactions between signaling ligands, receptors and their cofactors. Here, we used CellChat to calculate potential spot–spot interactions instead of cell–cell interactions and to infer spatial region–region communication. Briefly, we followed the official workflow and loaded normalized data of 8 slices into “createCellChat” function to create CellChat objects. Next, we loaded the CellChatDB.mouse ligand–receptor database and used “identifyOverExpressedGenes” and “identifyOverExpressedInteractions” function with default parameters to identify overexpressed signaling genes and ligand–receptor interactions (pairs) associated with each region. “ComputeCommunProb” and “computeCommunProbPathway” functions with default parameters were used to identify putative interaction pairs and pathways. “netAnalysis_computeCentrality” function was applied to the netP data to determine the senders and receivers. To compare the communication differences between young and old mouse brain regions, we used “mergeCellChat” to merge objects and “netVisual_bubble” functions to visualize ligand–receptor pairs.

### 2.14 qPCR

Four young (2 months old) and four old (24 months old) mice (strain C57BL/6J) were sacrificed according to the approved protocol. The isocortex, HPF, TH, HY, and MB from brain tissue were isolated to prepare RNAs. Prior to RNA extraction, brain region samples were homogenized using a pellet pestle mixer in pre‐cooled TBS buffer. Total RNAs were extracted using TRIZOL (Invitrogen). All RNA samples were subjected to DNase digestion, and 1 μg total RNA was used to reversed transcribed to cDNAs using HiScript III RT SuperMix for qPCR (Vazyme) following the manufacturer's instructions. cDNA concentrations were measured on a NanoPhotometer® N50 (Implen) and diluted to 100 ng/μL in DEPC‐treated water. Real‐time PCRs were done on Roche LightCycler 480IIusing the LightCycler® 480 SYBR Green I Maste. The oligonucleotide sequences used for qPCR in this study are listed in Table S3. And all the primers have the amplification efficiency close to 100%. Results were analyzed with the Roche–LightCycler® relating to CD software and the comparative CT method. Data are expressed as 2^−ΔΔCT^ for the experimental gene of interest normalized to the housekeeping gene (Gapdh) and presented as fold change relative to the average of control.

### 2.15 Immunohistochemistry

Mice were anesthetized and then perfused with PBS and 4% paraformaldehyde (PFA). Brain was dissected and fixed in 4% PFA at 4°C overnight. Then the brain was dehydrated with 10%, 20% and 30% sucrose in order. The dehydrated brain was mounted with O.C.T. compound and sectioned at 30 mm. After washed by PBS (three times, 15 min per time), the collected brain slices were permeabilized with 0.5% Triton‐X100 for 30 min at room temperature and the blocked with blocking buffer for 1 h. Next, the diluted primary antibodies (S100b, 1:200, ab52642, Abcam, UK; Wnt9a, 1:1000, ab125957, Abcam, UK; Wnt10a, 1:1000, ab106522, UK) were applied on brain slices at 4°C overnight. After incubation, brain slices were washed by PBS (four times, 10 min per time), and incubated with the secondary antibodies (Donkey anti rabbit 488, 1:500, 711‐545‐152, Jackson ImmunoResearch Laboratories Inc., US or Donkey anti rabbit Alexa Fluor™ 488, 1:500, A‐21206, ThermoFisher Scientific) for 2 h at room temperature, and then with DAPI for 5 min at room temperature. Brain slices were washed by PBS (four times, 10 min per time) and mounted with AQUA‐MOUNT (Thermo Scientific, US). Brain slices were scanned by VS200 (Olympus, Japan) and fluorescence imaging microscope (Eclipse Ni, Nikon Inc, Japan) and confocal microscope (LSM 880, ZEISS, Germany).

## 3 RESULTS

### 3.1 Identification of spatial regions of mouse brain

We captured the spatial patterns of gene expression, covering the cerebrum, brainstem and fiber tracts regions, from brain hemispheres in two young (2 months old) and two old (28 months old) mice using ST (Figure 1a and Table S1). After quality control (QC), a total of 27,089 spots and 19,797 unique genes were identified, with an average of 19,425 reads and 5243 genes per spot. The spots were distributed into 34 distinct clusters by unsupervised clustering. Uniform manifold approximation and projection (UMAP) dimensionality reduction was used to visualize the 34 identified clusters (C0‐C33) that showed the underlying molecular‐spatial structure (Figure 1b), ranging in size from 58 to 2826 spots and from on average 842 to 6454 genes, per cluster (Figure S1A,B).

To characterize these clusters, we identified the highly expressed genes in each cluster and aligned each H&E image to the Allen mouse brain reference atlas (ABA; www.brain‐map.org) (Figure 1c,d and Figure S1C). The isocortex‐related marker genes Stx1a, Stab2, and Pak7 were highly expressed in five clusters (C15, 12, 9, 4 and 6), the hippocampal formation (HPF)‐related marker genes Hpca, Cnn3 and Grp were expressed in seven clusters (C7, 13, 17, 24, 3, 21, and 23), the interbrain (IB)‐related genes Slc17a6, Plekhg1 and Esrrg were expressed in five clusters (C8, 28, 33, 10, 22), and the fiber tracts‐related gene Plp1 was expressed in C2 (Figure 1d). The spatial location of these clusters was confirmed by ABA (Figure 1c). In addition, we visualized the expression of the isocortex marker Stx1a, the HPF marker Hpca, the fiber tracts marker Plp1, the thalamus (TH) marker Prkcd, the hypothalamus (HY) marker Gpx3, and the midbrain (MB) marker Cd83 in brain sections of in our study (Figure 1e), which was also consistent with the ABA in situ hybridization (ISH) signal (Figure S1D). These results showed that the ST data could accurately partition the brain regions. Finally, integrating the cluster‐specific gene expression and spatial information (see Methods for detailed cluster naming rules), 27 spatial regions were defined (Figure 1f), where 22 regions appeared in multiple sets of slices (Figure S1E).

### 3.2 Spatial‐specific transcriptional changes in aging brain

**FIGURE 2:**
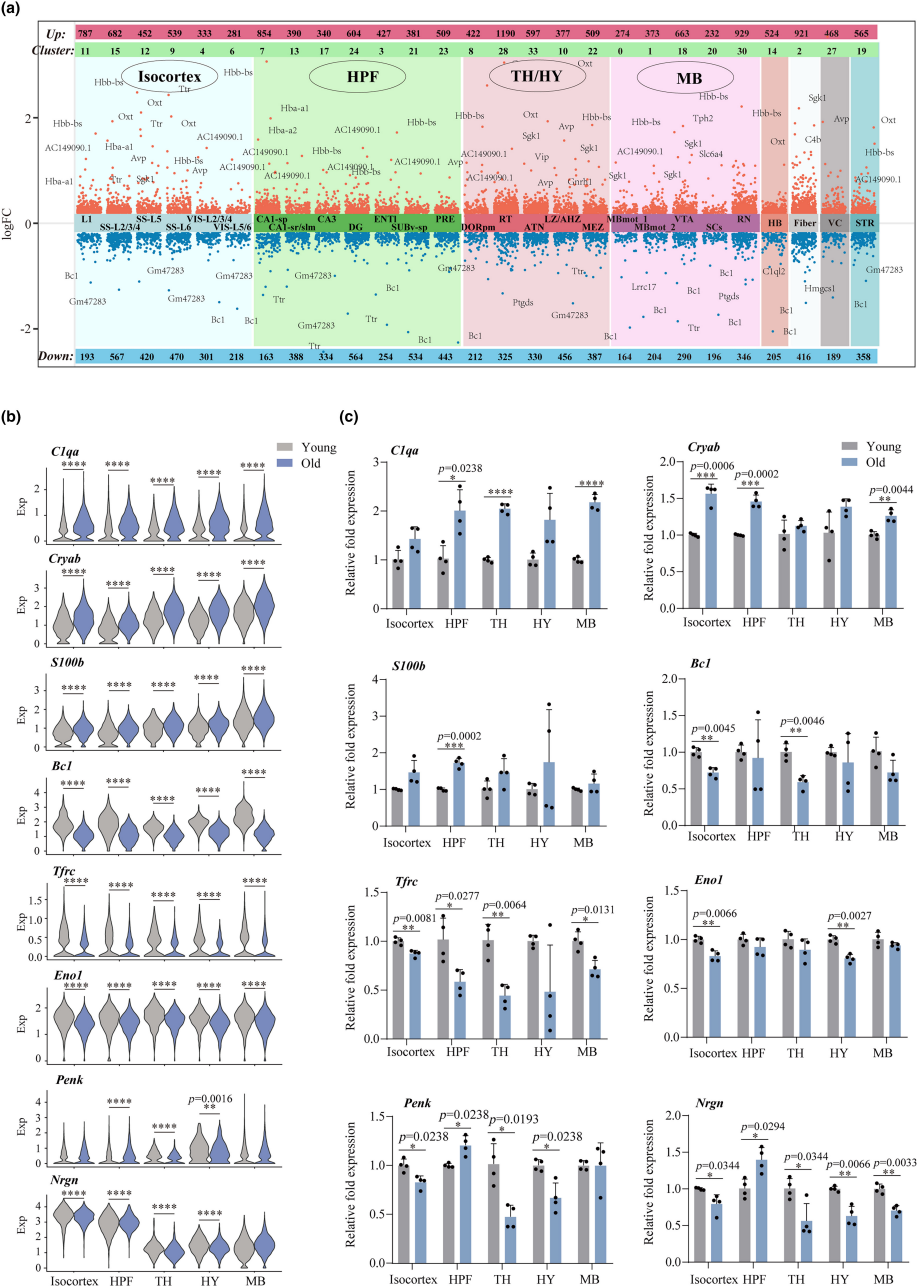
Spatial‐specific transcriptional changes in aging brain. (a) Scatter chart showing DEGs (dots) in 27 brain spatial domains. The red/blue dots show that gene expression increases/decreases with aging, and the top and bottom show the number of DEGs. (b) Violin plot showing gene expression differences of C1qa, Cryab, S100b, Bc1, Tfrc, Eno1, Penk and Nrgn in Isocortex, HPF, TH, HY and MB regions using spatial data; *p < 0.05, **p < 0.01, ***p < 0.001, ****p < 0.0001 by *t*‐test. (c) Histogram showing the relative fold expression of C1qa, Cryab, S100b, Bc1, Tfrc, Eno1, Penk and Nrgn in Isocortex, HPF, TH, HY and MB regions by qPCR; *p < 0.05, **p < 0.01, ***p < 0.001, ****p < 0.0001 by *t*‐test.

We next characterized transcriptional changes in the 27 brain spatial regions. A total of 5628 genes were significantly changed by aging in one or more regions (*p* < 0.05 and more than 10% of fold change (FC)) (Figure 2a). Some genes were upregulated in almost all the brain regions, such as C1qa, Cryab, and S100b, which have been reported to be strongly associated with brain aging (Lu et al., 2019; Stephan et al., 2013) (Figure 2b). Some genes were downregulated in almost all the brain regions, such as Bc1, a non‐protein‐coding RNA related to synaptic plasticity, learning and memory (Mus et al., 2007) and Tfrc, which has been shown to be downregulated in the hippocampus, frontoparietal cortex and corpus callosum of aged mouse (de Magalhaes et al., 2009; Soto et al., 2015) (Figure 2b). Interestingly, some genes showed opposite direction of changes in the different brain regions, such as Penk and Nrgn, which were upregulated in the HPF, but downregulated in the TH and HY (Figure 2b), indicating a spatial regulation pattern of genes with aging. Differences in gene expression may arise from aging‐related remodeling of neuronal subpopulations and signaling pathways between regions. To further experimentally validate the spatial‐specific changes of these genes, we conducted qPCR experiments on four young and four aging mice and obtained consistent results as shown in ST data (Figure 2c). For S100b, we used immunohistochemistry and found that multiple regions were also upregulated in old samples (Figure S2).

**FIGURE 3:**
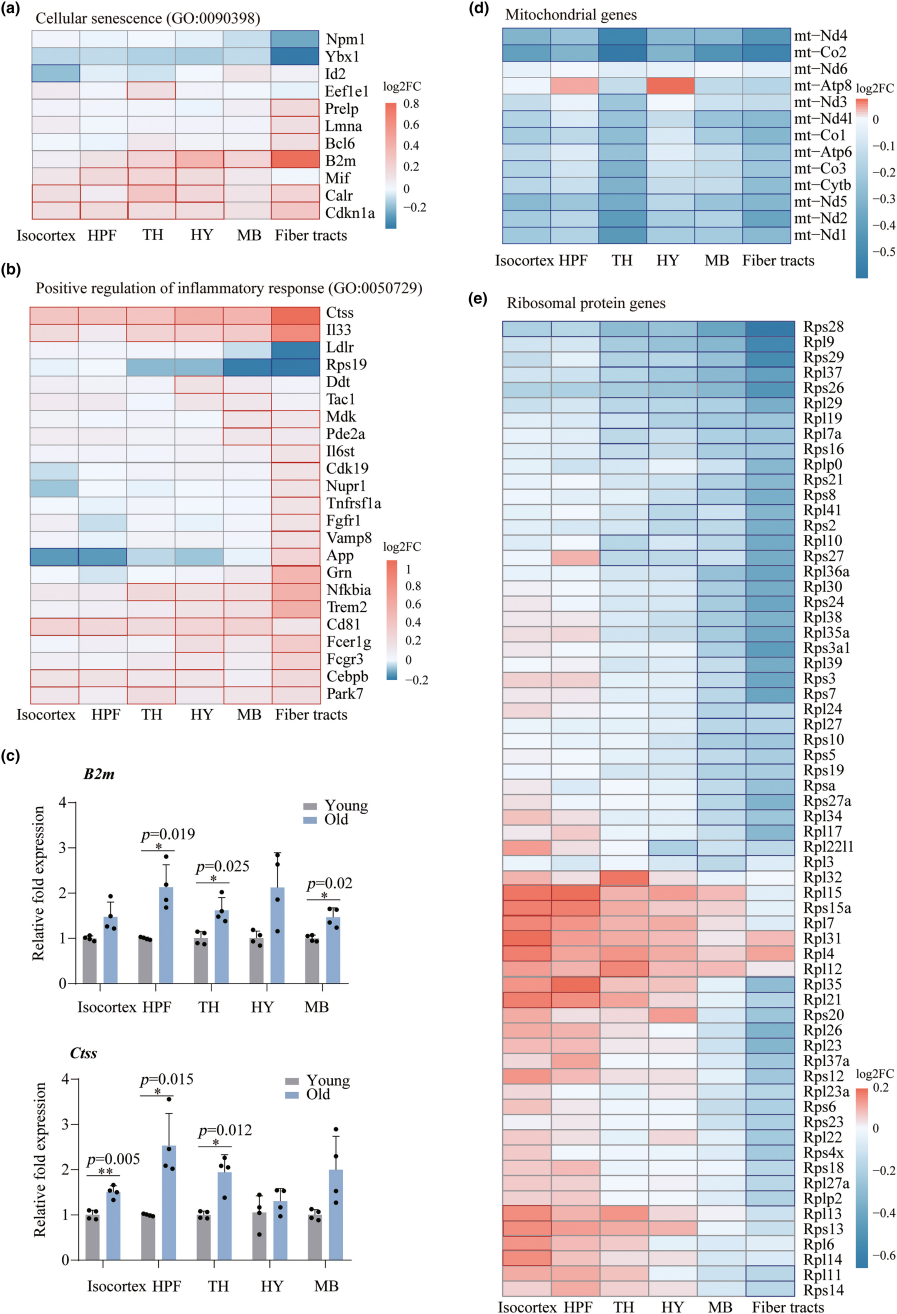
Spatial‐specific transcriptional changes in genes associated with cellular senescence and inflammation. (a) Heatmap showing cellular senescence‐related genes in 6 brain spatial domains. The red/blue box colors represent up/downregulation with aging, respectively. (b) Heatmap showing positive regulation of inflammatory response related genes in 6 brain spatial domains. The red/blue box colors represent up/downregulation with aging, respectively. (c) Histogram showing the relative fold expression of B2m and Ctss in Isocortex, HPF, TH, HY and MB regions by qPCR; *p < 0.05, **p < 0.01 by *t*‐test. (d, e) Heatmap showing mitochondrial genes and ribosomal protein genes in 6 brain spatial domains. The red/blue box colors represent up‐ and downregulation with aging, respectively.

We next examined some important gene groups which are known to play an important role in brain aging. Brain aging is known to associate with cellular senescence and inflammation (Chinta et al., 2015; Corlier et al., 2018; Sikora et al., 2021). We first specifically examined the transcriptional changes of genes related to cellular senescence. The gene group contains 87 genes annotated by Gene Ontology (GO:0090398), of which 11/25 changed in at least one big/sub‐region in our dataset (Figure 3a and Figure S3A, Table S2). Across all brain subregions, the proportion of up‐ and down‐regulated genes was 12% and 3.85%, respectively (Figure S3A,C). B2m, which is a systemic pro‐aging factor (Smith et al., 2015), as well as Mif, Calr and Cdkn1a, were significantly upregulated (*p* < 0.05), while Ybx1, Kras, and Npm1 were downregulated, in most the brain regions. Interestingly, Id2 and Ypel3 showed bidirectional patterns across different brain regions (Figure 3a and Figure S3A). Next, we analyzed the genes related to positive regulation of inflammatory response. This gene group contains 152 related genes annotated by GO:0050729, of which 23/39 are altered in at least one big/sub‐region in our dataset (Figure 3b and Figure S3B, Table S2). Across all brain regions, up to 21.8% of genes related to inflammation were upregulated (Figure S3B,C). Ctss, Cd81, and Il33, which were upregulated in almost all the brain regions, and only a few genes, such as Rps19 and App, were downregulated (Figure 3b and Figure S3B). We verified the aging‐related changes of B2m and Ctss in their corresponding brain regions (Figure 3c). These results showed that gene expression associated with cellular senescence and inflammation were primarily activated with aging across the brain regions, while some genes had different patterns of expression changes in the different subregions of the brain.

Mitochondria are known to be a key player in the aging process (Correia‐Melo et al., 2016; Haas, 2019). The expression changes of mitochondrial genes are unclear in various regions of the aging brain. Here, we explored the changes in the expression of 13 mitochondria‐encoding genes in 27 molecular regions and found that mitochondrial genes were generally down regulated in brain regions with aging (Figure 3d and Figure S3D), indicating mitochondrial dysfunction and dysregulated energy metabolism during aging. Notably, mitochondrial genes were upregulated in some specific regions, such as upregulation of mt‐Atp8 in the CA1, CA3, DG, and STR regions (Figure S3D), implying its specific biological role.

In addition, previous studies have shown that changes in ribosomal genes can lead to abnormal protein synthesis in aging (Gonskikh & Polacek, 2017; Kobayashi, 2014; Turi et al., 2019). Here, we found that the 64 ribosome protein genes showed region specific changes (Figure 3e). Ribosomal genes can be distinguished into three distinct patterns by examining the expression in brain regions. Some ribosomal protein genes, such as RPl12, Rpl4 and Rps15/Rps26, Rps28 and Rpl29, were up/downregulated across almost all the brain regions with aging, but some genes, such as Rpl11, Rpl17, and Rpl34, were upregulated in the isocortex, HPF and IB regions and downregulated in the MB, fiber tracts and VC regions (Figure 3e and Figure S3E), highlighting their potentially distinct responses to aging. It is notable that almost all the ribosome genes were upregulated in the STR region (Figure S3E).

In conclusion, we characterized spatial‐specific changes in gene expression in brain aging, particularly those changes in genes related to cellular senescence, inflammation, mitochondria, and ribosome. While we found while genes in these four functional categories generally had changes in gene expression consistent with prior knowledge, many genes showed spatial‐specific gene expression in the brain during aging, which provides important clues to understand genes with specific functions in different brain regions in aging.

### 3.3 Aging‐related transcriptional changes in the isocortex

**FIGURE 4:**
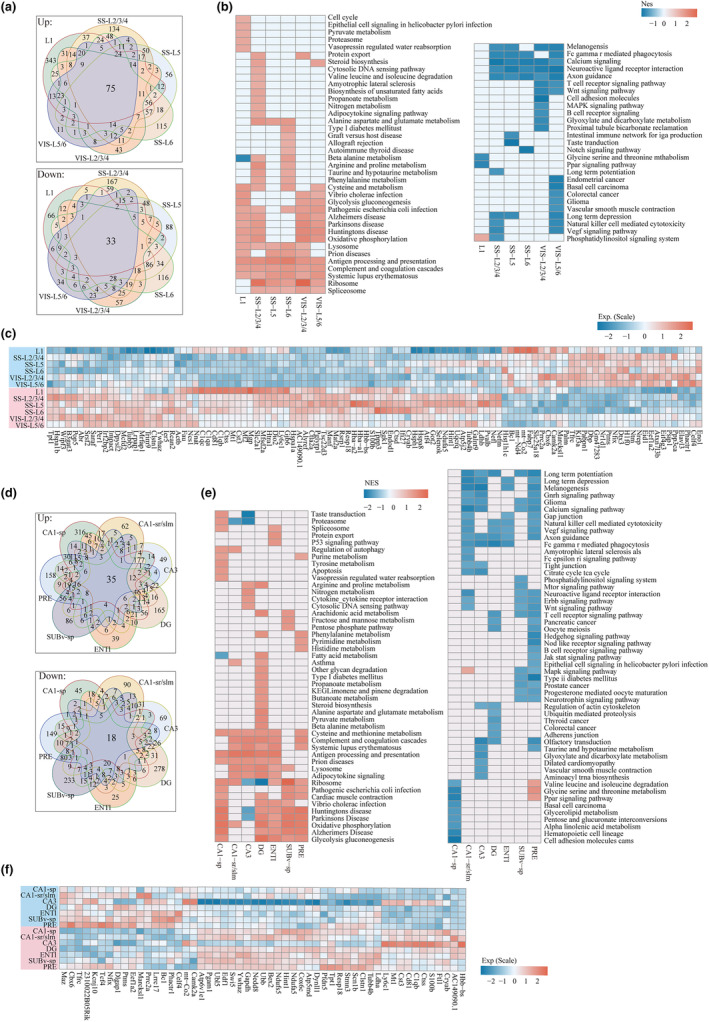
Aging‐related transcriptional changes in cerebral cortex subregions. (a) Venn diagram showing commonalities and differences between the DEGs of the isocortex subregions. (b) Heatmap showing aging‐related pathways (*p* < 0.05 and *q* < 0.25) across 6 subregions. The legend corresponds to the normalized enrichment scores (Nes), which represent the strength of the relationship between the phenotype and gene signature. Positive Nes values (red) indicate the pathways enriched in the old group, while negative Nes values (blue) indicate those enriched in the young group. (c) Heatmap showing both up/downregulated gene expression in the isocortex subregions. (d) Venn diagram showing commonalities and differences between the DEGs of HPF subregions. (e) Heatmap showing aging‐related pathways (*p* < 0.05 and *q* < 0.25) across HPF subregions. (f) Heatmap showing both up/downregulated gene expression in the HPF subregions.

We next investigate transcriptional changes during aging in the specific brain regions as well as their subregions. In the isocortex, we identified six subregions, named L1, SS‐L2/3/4, SS‐L5, SS‐L6, VIS‐L2/3/4 and VIS‐L5/6, based on molecular expression. By comparing the differentially expressed genes (DEGs) during aging in each subregion, we found 1413 DEGs in at least one subregion. Most of DEGs were subregion‐specific, of which only 108 genes exhibited the same directionality in all the subregions of the whole isocortex region (75 upregulated and 33 downregulated) (Figure 4a,c), including S100b (Tiu et al., 2000), Ctss (Park et al., 2009) and C1q family (Stephan et al., 2013), which were previously identified as aging‐related genes, and some previously unreported genes related to subregional aging, such as Rab3a (L1), Kdm5d (SS‐L2/3/4), Cebpd (SS‐L5), Dlk1(SS‐L6), Atp5h (VIS‐L2/3/4), and Psd (VIS‐L5/6).

To further understand function relevance of these DEGs, we analyzed changes in the Kyoto Encyclopedia of Genes and Genomes (KEGG) pathways in each subregion during aging by performing gene set enrichment analysis (GSEA). We found 65 aging‐related pathways (*p* < 0.05) across the examined subregions in shared or subregion‐specific manners (Figure 4b). Of those pathways, 15 pathways were activated, and 6 pathways were inhibited in at least 3 subregions. As expected, aging‐related pathways, such as AD, Parkinson's (PD), and Huntington's diseases (HD), were activated. In contrast, Fc gamma r‐mediated phagocytosis and calcium signaling were inhibited in major subregions, consistent with previous studies (Chandran et al., 2019; Fu et al., 2015; Park et al., 2020). In addition to these known pathways, antigen processing and presentation, complement and coagulation cascades and systemic lupus erythematosus were activated in all the subregions. Notably, in VIS−L2/3/4, cell adhesion molecules, the MAPK signaling pathway, B‐cell receptor signaling, glyoxylate and dicarboxylate metabolism and proximal tubule bicarbonate reclamation were downregulated, while in VIS‐L5/6, some cancer‐related pathways, such as endometrial cancer, basal cell carcinoma, colorectal cancer and glioma, were inhibited (Figure 4b), indicating that different cortical layers have great functional differences with aging.

### 3.4 Aging‐related transcriptional changes in the HPF

In the HPF, seven subregions were identified, named CA1‐sp, CA1‐sr/slm, CA3, DG, ENTI, SUBv‐sp and PRE. HPF plays a critically important role in memory and learning (Jaffard & Meunier, 1993), which is consistent with our study that the highly expressed genes in all the HPF subregions were enriched in learning or memory, cognition and axon development (Figure S4A). Next, we also compared the DEGs of each HPF subregion during aging, we identified 2946 DEGs, of which 53 genes (35 upregulated and 18 downregulated) showed consistent spatial patterns (Figure 4d,f), such as AC149090.1, Cryab and Ubb. Each subregion also identified some self‐related aging genes, including 45 genes in CA1‐sp, 90 in CA1‐sr/slm, 69 in CA3, 278 in DG, 25 in ENTI, 233 in SUBv‐sp and 149 in PRE (Figure 4f).

GSEA identified 100 significant pathways in the HPF subregions (*p* < 0.05) (Figure 4e). Aging‐related pathways, such as AD, PD, and HD, were also activated in almost all HPF regions. Interestingly, PD and HD pathways were inhibited in CA3 regions, indicating that not all regions equally contribute to neurodegeneration with aging. This difference may be due to susceptibility to protein aggregation or glial dysfunction. In addition, antigen processing and presentation, complement and coagulation cascades, oxidative phosphorylation and glycolysis and gluconeogenesis were also activated in most subregions (Figure 4e). In contrast, some key pathways, such as long‐term potentiation (LTP), the calcium signaling pathway, and the VEGF signaling pathway, were suppressed in most subregions. Notably, LTP is a long‐lasting increase in synaptic efficacy and is the molecular basis for learning and memory (Martinez Jr. & Derrick, 1996). We further analyzed the learning or memory function terms containing 319 genes, of which 112 genes were differentially expressed in the subregions (Figure S4B). Some genes, such as S100b and Atp1a3, were differentially expressed in multiple regions, suggesting that the HPF subregions coordinately regulate learning and memory functions.

### 3.5 Aging‐related transcriptional changes in the brainstem and fiber tracts

Previous studies indicated early brainstem alterations in a myriad of neurodegenerative diseases and dementias (Bouhrara et al., 2020) and more recent findings indicated that aging was associated with glial senescence in the brainstem (Balasubramanian et al., 2021). However, there is no systematic investigations addressed aging‐dependent differences in the brainstem region as well as the subregions, despite its important role in the regulation of brain functions.

**FIGURE 5:**
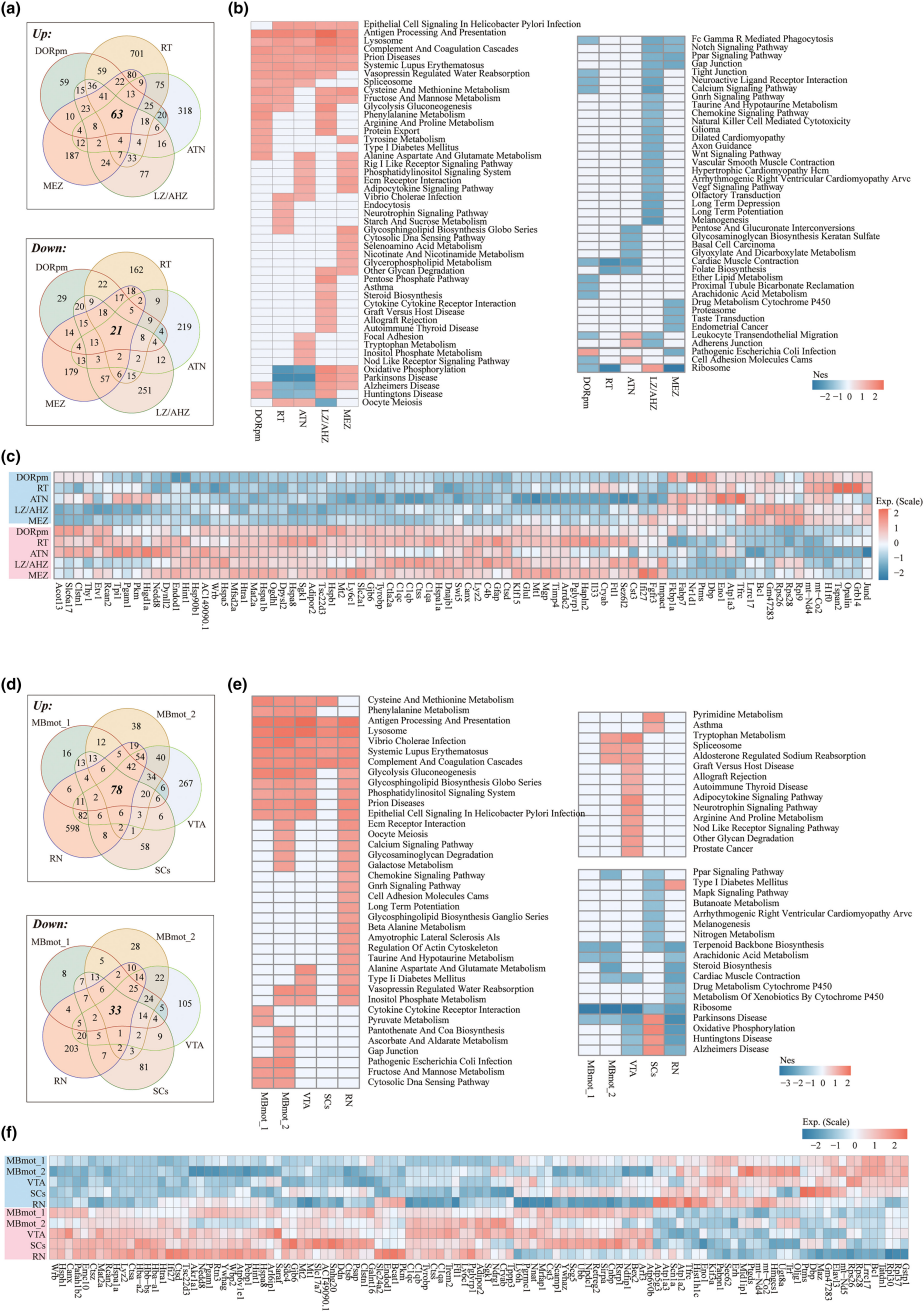
Aging‐related transcriptional changes in brainstem subregions. (a) Venn diagram showing commonalities and differences between the DEGs of IB subregions (TH and HY). (b) Heatmap showing aging‐related pathways (*p* < 0.05) across interbrain subregions. (c) Heatmap showing both up/downregulated gene expression in the IB subregions. (d) Venn diagram showing commonalities and differences between the DEGs of MB subregions. (e) Heatmap showing aging‐related pathways (*p* < 0.05) across midbrain subregions. (f) Heatmap showing both up/downregulated gene expression in the MB subregions.

We identified three thalamic (TH) regions (DORpm, RT and ATN) and two hypothalamic (HY) regions (LZ/AHZ and MEZ) in the brainstem region. 63 and 21 genes were up‐ and downregulated in these regions, respectively (Figure 5a,c). GSEA showed that during aging, the enriched pathways in the TH and HY included activation of antigen processing and presentation, lysosomes, complement and coagulation cascades, prion diseases, and systemic lupus erythematosus. Interestingly, aging‐related neurodegenerative diseases, such as AD, PD, and HD, were activated in the HY region but inhibited in the RT and ATN thalamic regions (Figure 5b). We also identified five subregions (MBmot_1, MBmot_2, VTA, SCs, and RN) of the MB. There are 78 and 33 consistently up‐ and downregulated genes in the MB region, respectively (Figure 5d,f). The main activation pathways of the MB were antigen processing and presentation and lysosomes, which was also found in the TH and HY regions. In addition, each subregion also has its own unique functional changes. For example, the VTA region activates autoimmune thyroid disease and the adipocytokine signaling pathway, but the RN activates the chemokine signaling pathway and the Gnrh signaling pathway, and SCs activate the neurodegenerative disease‐related pathway (Figure 5e).

Fiber tracts (white matter) are mainly responsible for the exchange of signals between various parts of the brain, but little is known about the gene expression and functional changes in fiber tracts with aging. Consistent with the known physiological function of fiber tracts, our enrichment analysis of highly expressed genes in fiber tracts were enriched in the regulation of membrane potential, neurotransmitter transport, neurotransmitter secretion and signal release from synapses (Figure S5A). We next identified 102 upregulated and 78 downregulated genes in the fiber tracts region during aging (Figure S5B,C). KEGG pathway enrichment analysis showed that the upregulated genes of fiber tracts in the aging process were enriched not only in neurodegenerative diseases such as AD, PD, and HD but also in lysosomes, phagosomes and apoptosis (Figure S5D). GO analysis indicated that upregulated genes were enriched in the ATP metabolic process, antigen processing and presentation of exogenous peptide antigen, and antigen generation of precursor metabolites and energy (Figure S5D). These results indicated the important role of fiber tracts in the aging process, in which apoptosis and phagocytosis may play an important role.

### 3.6 Cell type distributions in the brain regions

**FIGURE 6:**
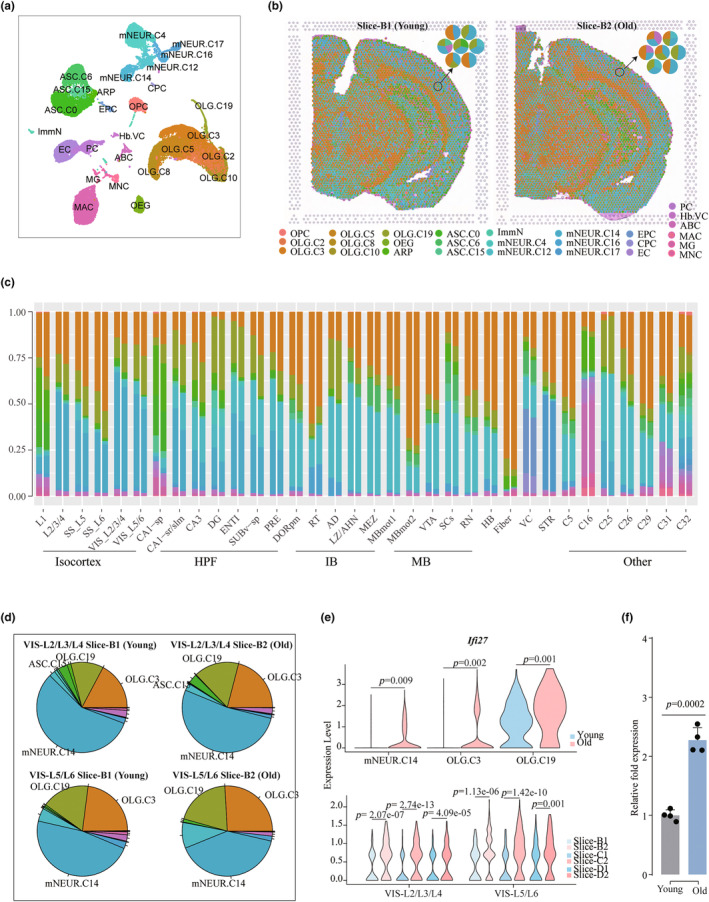
Integrating spatial transcriptomics and single‐cell RNA‐seq. (a) UMAP plot of all cells, colored by cell type; *n* = 37,096 individual cells. (b) Spatial distribution of cell types. Left and right are the distributions on slice B1 (young) and slice B2 (old), respectively. (c) Histogram showing the proportion of cell types of spots contained in each spatial domain in young and old mice. (d) Pie chart showing the proportion of cell types contained in the VIS‐L2/3/4 and VIS‐L5/6 regions in slice B. (e) Violins showing the expression of Ifi27 in cell types (top) and regions (bottom), the *p*‐value is calculated by *t*‐test. (f) Relative fold expression of Ifi27 in Isocortex by qPRC, the *p*‐value is calculated by *t*‐test.

We next sought to understand changes of cell types in specific brain regions during aging. The mammalian brain is known to have extremely complex cell types (Ximerakis et al., 2019). To data, cellular distribution in different brain regions during aging is not clearly understood. To this end, we characterize cell types in each spot on the brain slice by combining our ST datasets with single‐cell reference datasets (GSE129788) (Ximerakis et al., 2019) that annotated 27 cell types (Methods, Figure 6a and Figure S6A,B). We estimated proportions of each cell type in each individual region (Figure 6b and Figure S7A). Consistent with the known knowledge, we found that the isocortex and the brain stem (IB, MB and HB) were mainly composed of mature neurons (mNEUR), oligodendrocytes (OLG) and a small number of astrocytes (ASC), the fiber tracts area was mainly composed of OLGs and the VC area contained a large number of EC cells (Figure 6c) (Hofmann et al., 2017). Next, we compared the proportion of cell components contained in each brain region between the young and old mice. We could see clear changes in the cell compositions during the aging process. For example, in the VIS area (VIS‐L2/3/4 and VIS‐L5/6), OLG.C3 and OLG.C9 cells were increased, and the corresponding mNeur.C14 cells were decreased with aging (Figure 6d and Figure S7B). Notably, we found that a gene, Ifi27, an interferon α‐inducible protein, was upregulated in both the mNEUR.C14, OLG.C3 and OLG.C9 cell types and in the VIS‐L2/3/4 and VIS‐L5/6 areas during aging (Figure 6e). We further verified that Ifi27 is upregulated in the VIS by qPCR (Figure 6f). A previous study also showed that the innate immunity‐related Ifi27 gene was upregulated in aging brain endothelial cells (Chen et al., 2020). Therefore, Ifi27 might be a biomarker of aging in the VIS region.

During aging, the number of new nerve cells in the brain gradually decreases, resulting in a decline in brain function (Lee et al., 2012; Nicaise et al., 2020). Therefore, we detected the distribution of immature neurons (ImmN), which are the source of new neurons in the brain. Interestingly, we found that in the brains of young and old mice, ImmN are mainly concentrated in the DG region of the hippocampus (Figure S8A), which is consistent with some previous findings (Bruel‐Jungerman et al., 2005; Mandyam et al., 2018; Vivar & van Praag, 2013). Furthermore, we calculated the proportion of cell types contained in the DG region and found that the proportion of ImmN decreased during aging (Figure S8B). Comparing the DG region between old and young samples, upregulated genes were mainly enriched in various metabolic processes, such as the generation of precursor metabolites and energy and ATP metabolic processes, while downregulated genes were enriched in axon development, cell projection organization and dendrite morphogenesis (Figure S8C). These results indicate that during the aging process, the formation of new neurons is blocked in the DG region.

### 3.7 Changes in communications between spatial regions during aging

The brain is a collection of about 10 billion interconnected neurons, and selective communication among different brain regions is crucial for brain function (Laughlin & Sejnowski, 2003; Schipul et al., 2011). Disruption of interconnected brain networks during aging might result in impaired connectivity between or within regions. Here, we explored how aging‐driven gene expression changes affect communication between brain regions based on ST data by CellChat (Jin et al., 2021).

**FIGURE 7:**
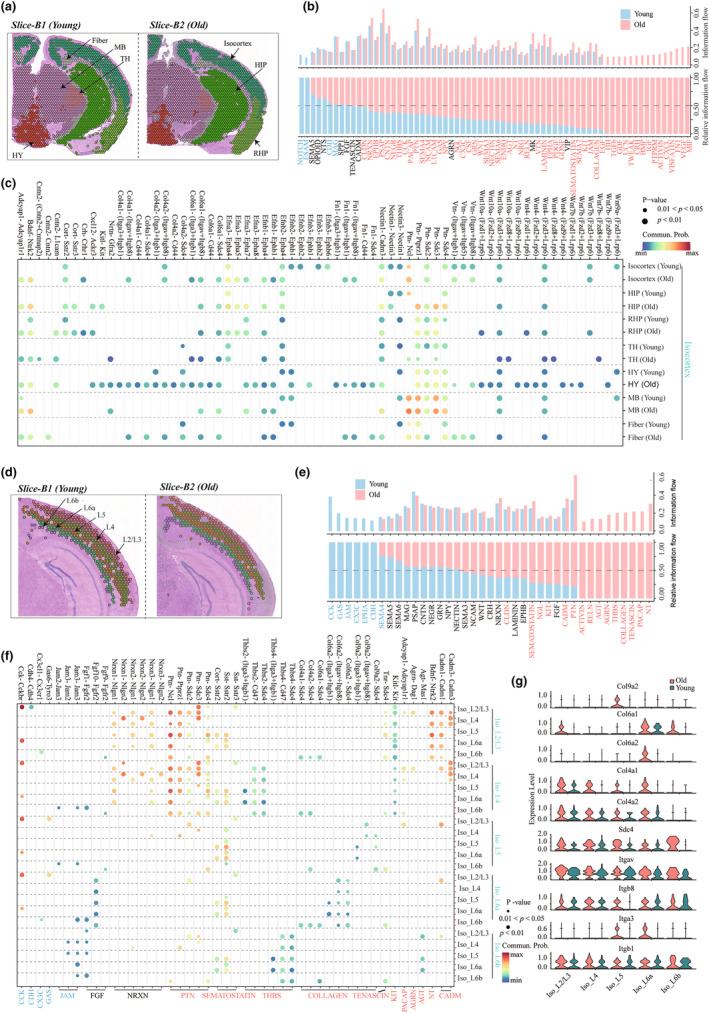
Communication between different regions based on spatial transcriptional maps. (a) The large regions were identified in the slices (slice‐B1 and slice‐B2), which mainly include the iscortex, HPF, TH, HY, MB, and fiber tracts. (b) All the significant signaling pathways were ranked based on their differences in overall information flow within the inferred networks between slice‐B from young and old mice. The overall information flow of a signaling network is calculated by summarizing all the communication probabilities in that network. The signaling pathways colored red were more enriched in old individuals, and those colored blue were more enriched in young individuals. (c) Comparison of the significant ligand–receptor pairs between slice B from young and old. The dot color reflects communication probabilities, and the dot size represents the computed *p* values. Empty space means the communication probability is zero. The *p* values were computed from a one‐sided permutation test. (d) Distribution of the isocortex subregions on slices (slice‐B1 and slice‐B2). (e) All the significant signaling pathways were ranked based on their differences in overall information flow within the inferred networks between isocortex subregions in young and old mice. (f) Comparison of the significant ligand–receptor pairs between isocortex subregions in young (slice‐B1) and old mice (slice‐B2). (g) Violin plot showing COLLAGEN signal‐related ligand receptor gene expression in different subregions from slice‐B.

We prioritized the calculation of the information interaction between large regions in young and old mice (Methods, Figure 7a and Figure S9A). During the aging process, some pathways become dysregulated, including the classic TGFb and WNT signaling pathways, which were seen in the four sets of slices in our ST datasets (Figure 7b and Figure S9B). We further analyzed the changes driven by pathway‐related ligand‐receptor modulation with aging and found that some ligand–receptor pairs not only were brain region‐specific and were only expressed in old mice (Figure 7c and Figure S11). For example, the ligand–receptor pairs related to dysregulated WNT signaling are mainly concentrated in the isocortex and HY regions (Figure 7c and Figure S11). Previous studies have shown that the aging process involves dysregulation of the WNT signaling pathway (Inestrosa et al., 2020). Our further analysis found that WNT‐related ligand receptor genes were not only regionally specific, but also differentially expressed with aging (Figure 7c and Figure S9C), The ligands Wnt10a and Wnt9a are expressed in the isocortex, RHP and TH region of old mice (Figure S10A), but not in young mice, which is further confirmed at the protein levels by using immunofluorescence staining (Figure S10B). These results suggest that WNT may disrupt synaptic integrity or neuronal integrity in a position‐dependent manner. Interestingly, we found that inflammation‐related pathways involving ligand–receptor pairs were also widely activated during aging, such as MIF (Mif‐Ackr3), IGF (Igf2‐Igf2r), EGF (Hbegf‐Erbb4), GDF (Gdf11‐(Tgfbr1 + Acvr2a)) and CXCL (Cxcl12‐ Ackr3) signals (Figure 7b,c), supporting the association of brain aging and inflammatory response.

The mammalian isocortex, containing six‐layered cortex, is involved in higher processes, including sensory perception, generation of motor commands, spatial reasoning, conscious thought, and language (Lodato & Arlotta, 2015; Lui et al., 2011). To accurately explore the interaction between the different layers in the isocortex that contains the VIS region, we further clustered the isocortex to classify the spots into L2/3, L4, L5, L6a, and L6b (Figure 7d and Figure S12A). Compared with the young, the isocortex regional information interaction changed significantly in the old brain, such as the obtained NT signal (Bdnf‐Ntrk2) and CADM signal (Cadm1‐Cadm1), mainly mediated by L2/3, and the COLLAGEN signal, mainly mediated by L6a, and the THBS signal, mediated by L4 and L6b (Figure 7e,f and Figure S12B). For the COLLAGEN signal, the ligands Col9a2, Col6a1, Col6a2, Col4a1, and Col4a2 are mainly expressed in aging, and the receptors Sdc4, Itgav, Itgb8, Itga3, and Itgb1 are also found to be more highly expressed in aging, indicating that this signal is indeed activated in the VIS region with aging (Figure 7g and Figure S13). Altogether, by leveraging the transcriptional profiles of each spot, we deciphered ligand‐receptor interactions involving pathways among nearly all the identified brain regions and identified potential information interaction between regions with aging.

## 4 DISCUSSION

In this study, we reported spatially‐resolved transcriptome of young and old mouse brains. We identified the brain regions based on molecular definitions that are not exactly same as the structural regions defined by ABA. Some large or special structure domains can be directly recognized, such as the STR, fiber tract and DG. For some more complex regions, such as the isocortex, we identified specific subregions (layers) with distinguishing molecular profiles. Notably, we also obtained the expression profiles of some small regions, such as C30 (red nucleus [RN]) and C33 (anterodorsal nucleus [AD]), which were previously difficult to obtain for quantitative evaluation and statistical analysis due to technical and methodological limitations.

We compared the transcriptome changes of 27 molecular regions during aging. We found that DEGs were widespread across all brain areas, some with a brain region‐specific manner. However, despite this, the aging mechanism among different brain regions was largely convergent because some genes were uniformly differentially expressed in multiple regions, and GSEA showed that almost all regions were related to the activation of neurodegenerative diseases and antigen processing and presentation. Surprisingly, more DEGs were tissue specific, indicating that different regions play different roles in the aging process. For example, the hippocampal subregions are more involved in long‐term potentiation (LTP) inactivation during aging, which has been known to be related to learning and memory (Barnes, 2003). Because of the limited previous knowledge of molecular regions, only a small fraction of the DEGs obtained here have been previously associated with brain aging. We believe that global spatial gene expression changes leading to functional differences can provide a new perspective for future aging research.

Our work further characterized the spatial distribution of cell types in the brain and the changes in the aging state. Ximerakis et al. (2019) identified aging‐related changes in nearly all mouse brain cell types and revealed different patterns of aging across different populations. However, the lack of spatial information makes it impossible to locate the spatial distribution of cell types. Here, combining the single‐cell transcription profile and the spatial transcriptional profile, we identified the cell types contained in each spot and found that the cell types are distributed in the brain with spatial characteristics. For example, OLG and mNeur cells were distributed in the VIS area, and the proportion of OLG increased and the proportion of OLG decreased during aging; ImmN were mainly concentrated in the DG region, and the proportion decreased during aging. Unfortunately, because the spatial transcriptome platform, 10X Visisum, has not reached the single‐cell level, and it is difficult to obtain a true single‐cell spatial expression profile. Even if combined with single‐cell transcription profiles, it is impossible to explore the expression changes of specific cell types in specific regions. This will be one of our future research directions.

Brain aging is a complex process that relies on the precise regulation of multiple brain regions (Kang et al., 2011). Unravelling how the brain regions communicate and change during the aging state is essential for understanding brain aging. Similar to the sending and receiving of electrical signals between brain regions (Lovinger, 2008), we used ligand–receptor interaction information to construct a gene interaction network in the young and old brain. We have unearthed some pathways that are affected by aging. For example, the isocortex regulates the other regions through the WNT signal, which is activated in the aging state. We do not know which pathway or ligand–receptor pair is more important in the aging process, but changes in global information interactions can provide a basis for future brain molecular interaction research.

It should be noted that the present study has some limitations. The first limitation is that the sequencing data came from only two young and two old mice separately, which may affect the reliability of the results. Only four spatial transcriptome slices were taken from each group, which limited our identification of some small regions in the brain. In addition, the samples we selected were all male mice, which prevented us from exploring the impact of gender on spatial gene expression changes during aging. Going forward, additional biological replicates, spanning both sexes and more brain regions, will be important to fully capture the complexity of aging‐related transcriptional dynamics. Emerging spatial omics techniques that provide true single‐cell resolution would also enable further exploration of specific cell types in distinct brain regions during aging. Despite these limitations in sample size and resolution, this work establishes an important spatial transcriptional map during brain aging that provides an important resource of spatially changed gene expression, cell types and information interactions. Our work facilitates the understanding of the aging process and provides biological insights into the molecular mechanism of brain aging.

## AUTHOR CONTRIBUTIONS

Z.X. conceived and supervised the study. Z.X. and C.W. designed the study. C.W. analyzed the data. Y.T.W., B.Y. and J.Y.Z. prepared materials for spatial transcriptome. C.W. and Z.X. interpreted data. T.X.T., Y.T.W. and X.L.Z. performed the experiments. M.Z.X. and C.W. constructed the website. C.W., and Z.X. wrote the manuscript. All authors read and approved the manuscript.

## FUNDING INFORMATION

This project was supported by National Key R&D Program of China (2021YFF1200903, Z.X.), Guangzhou Basic and Applied Basic Research Foundation (202201020336, Z.X.), National Natural Science Foundation of China (82171404, X.L.Z.), The Key Research and Development Program of the Ministry of Science and Technology (2022YFF1202901, X.L.Z.), Natural Science Foundation of Guangdong Province (2023A1515011529, X.L.Z.), Ministry of Science and Technology, P.R.China (2022ZD0208604 and 2022ZD0208605 to J.Z.), National Natural Science Foundation of China (31771195, 81790640, and 820712002 to J.Z.).

## CONFLICT OF INTEREST STATEMENT

All authors declare no competing financial interests.

## DATA CITATION

https://www.ncbi.nlm.nih.gov/geo/query/acc.cgi?&acc=GSE193107.

## Supporting information


Data S1:

## Data Availability

The spatial transcriptomic datasets were deposited in the GEO database (accession number: GSE193107). The entire dataset can be explored interactively at http://sysbio.gzzoc.com/Mouse‐Brain‐Aging/.
