# Supplementary material for: Spatially resolved transcriptome of the aging mouse brain
Source: Aging Cell. 2024 Feb 19;23(5):e14109. doi: 10.1111/acel.14109 (PMC11113349; doi:10.1111/acel.14109)
Supplement: Supplementary file 1 — Data S1: [file ACEL-23-e14109-s001.docx]

**Supplementary Information**

# Spatially resolved transcriptome of the aging mouse brain


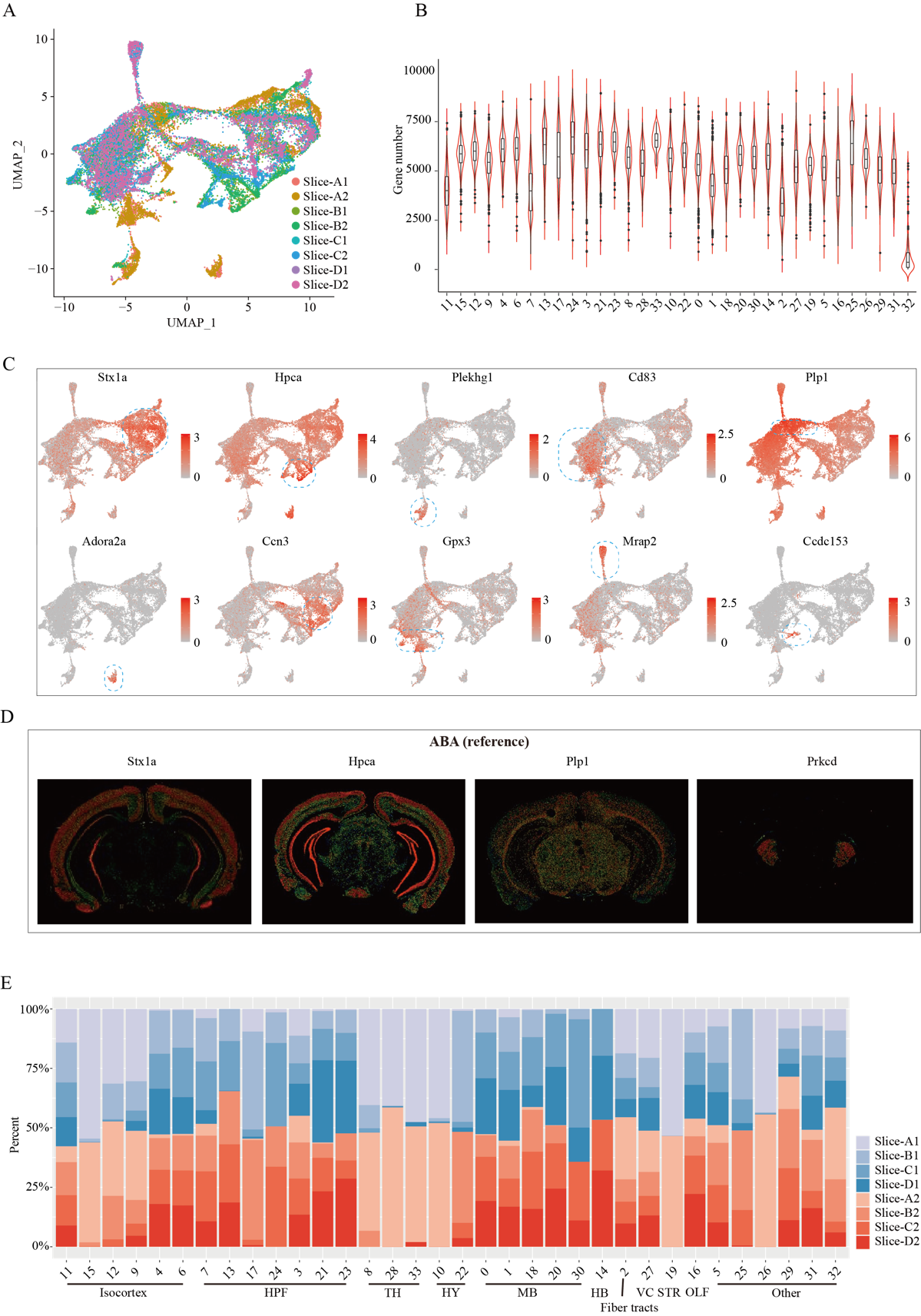


**Figure S1.** (A) U-MAP visualization of spots, colored by samples. (B) The box plot shows the number of genes in each cluster. (C) Spots colored by expression of large brain regions related marker genes (Isocortex: Stx1a; HIP: Hpca; RHP: Ccn3; TH: Plekhg1; HY: Gpx3; MB: CD83; HB: Mrap2; Fiber tracts: Plp1; STR: Adora2a; VC: Ccdc153). (D). Expression of Stx1a, Hpca, Plp1, Prked in ABA reference. (E). Bar chart for the proportion of samples in each cluster.

**
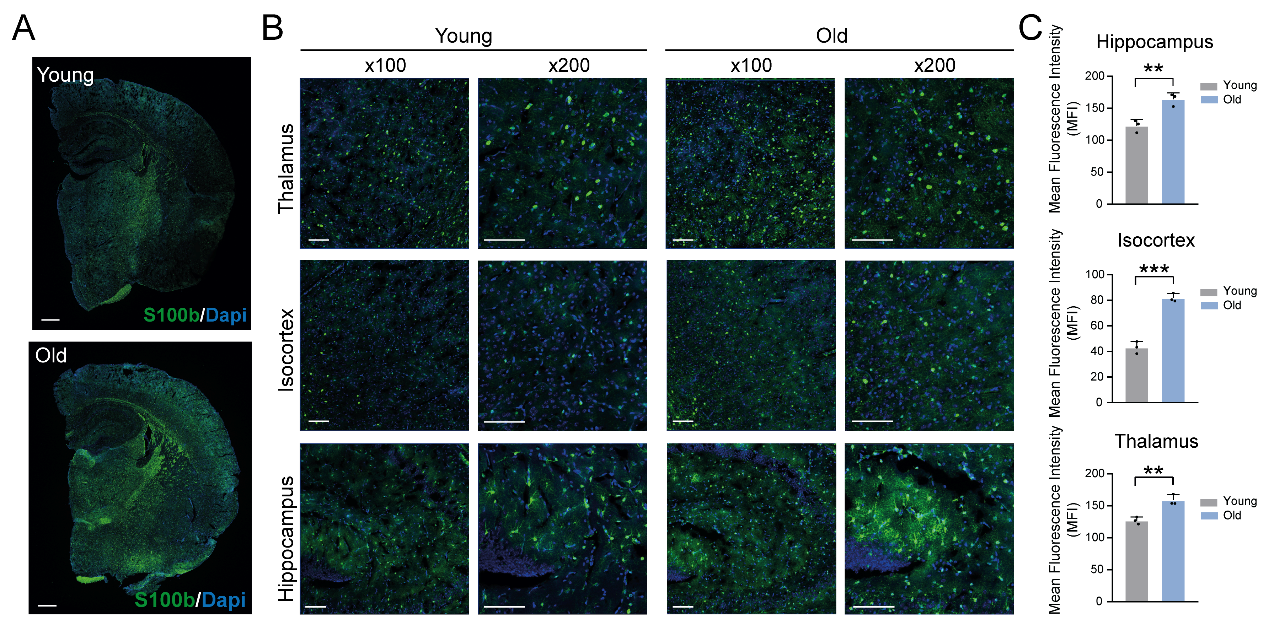
**

**Figure S2. (A)** Overview image of mouse brains stained for S100b (green). Scale bars = 500 µm. **(B)** Higher magnification images of representative region in mouse brain tissue. Scale bars = 100 µm. **(C)** Quantification of fluorescence intensity of S100b in different brain regions of young and old mice. ** p < 0.01, *** p < 0.001 by t-test.


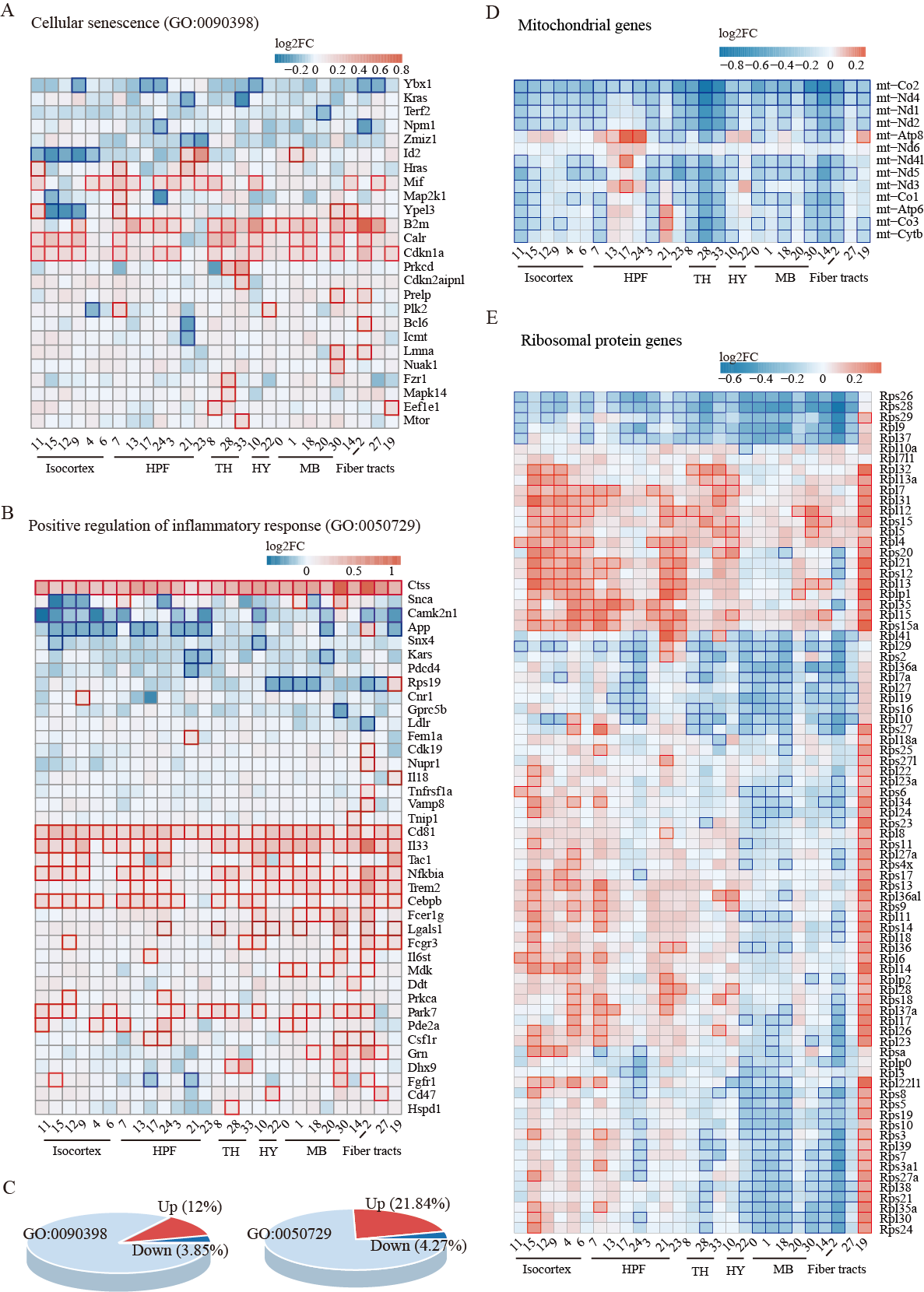


**Figure S3**. (A) Heatmap showing cellular senescence-related genes in 27 brain spatial domains. The red/blue box colors represent up/downregulation with aging, respectively. (B) Heatmap showing positive regulation of inflammatory response related genes in 27 brain spatial domains. (C) Pie chart showing the proportion of significantly up- and down-regulated genes across 27 brain spatial domains. (D, E) Heatmap showing mitochondrial genes and ribosomal protein genes in 27 brain spatial domains. The red/blue box colors represent up- and downregulation with aging, respectively.


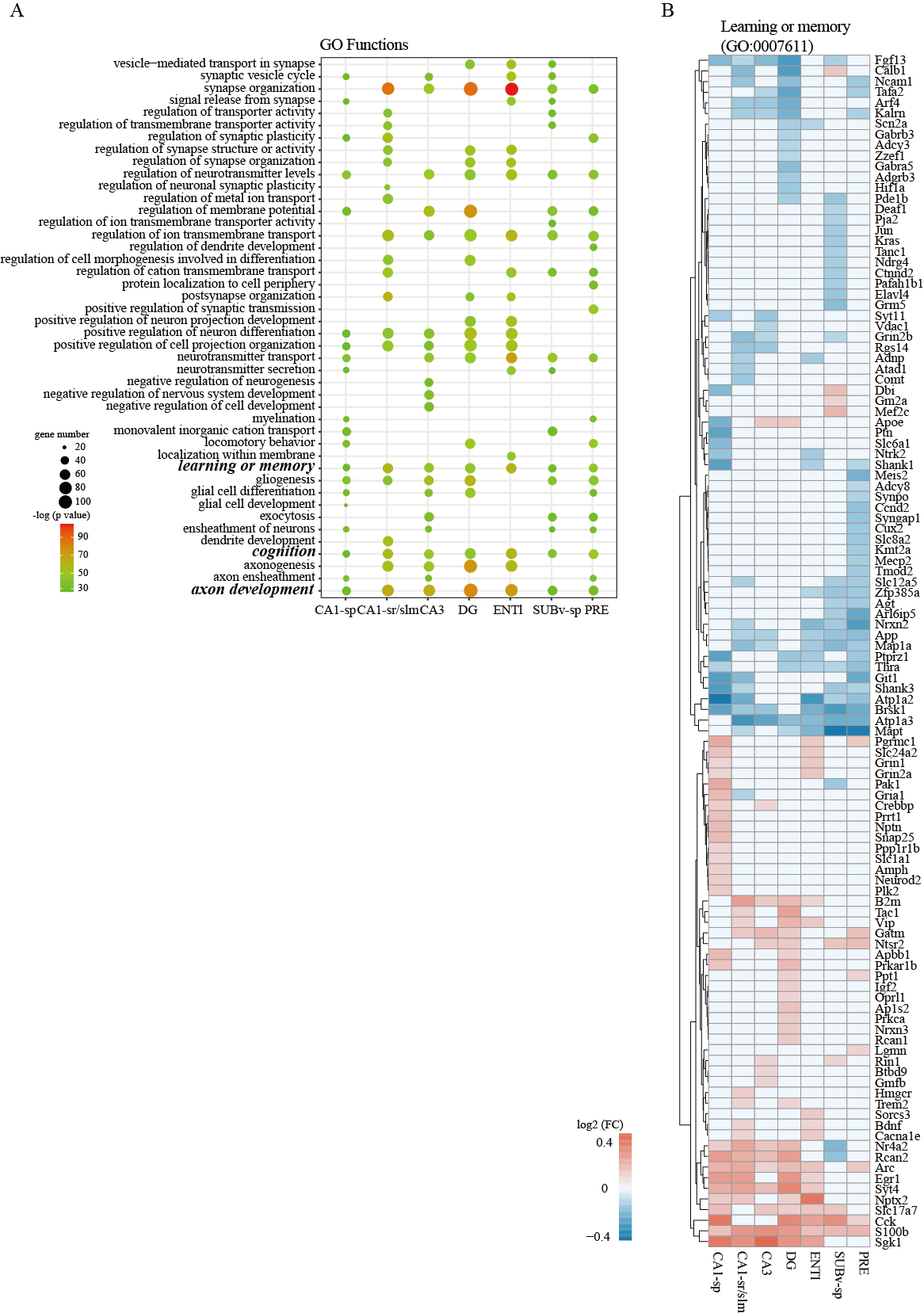


**Figure S4.** (A) The bubble plot showing the top 20 terms for the GO enrichment function of the highly expressed genes of the HPF subregions. (B) Heatmap showing a subset of learn or memory function related genes, red/blue is up-/down- regulation.


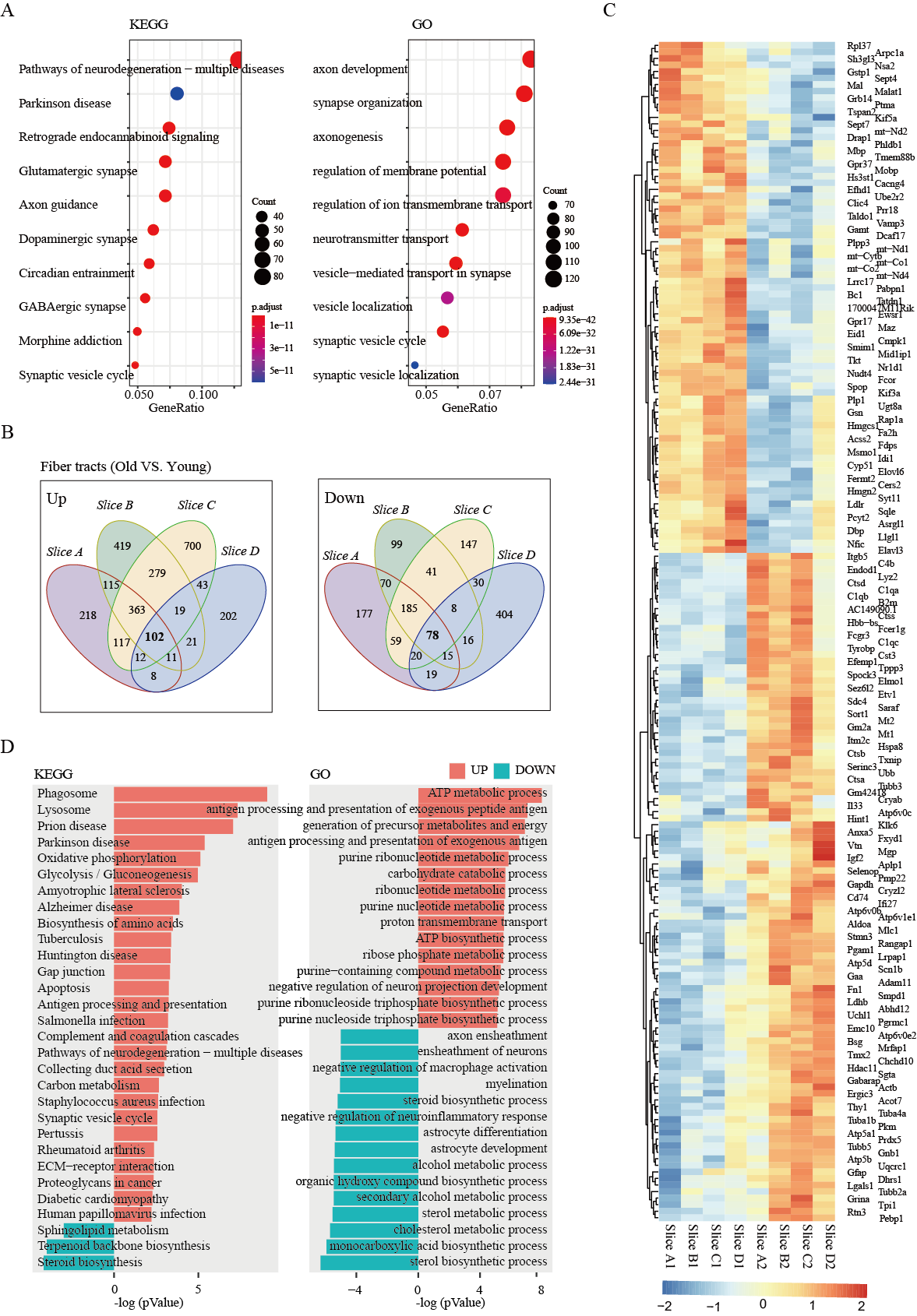


**Figure S5.** (A) KEGG and GO enrichment analysis of fiber tracts highly expressed genes. **(B)** Venn diagram showing commonalities and differences between the DEGs of fiber tracts in 4 group slices. (C) Heatmap showing DEGs in the fiber tracts region with aging. (D) KEGG and GO enrichment analysis of up/downregulated DEG genes.


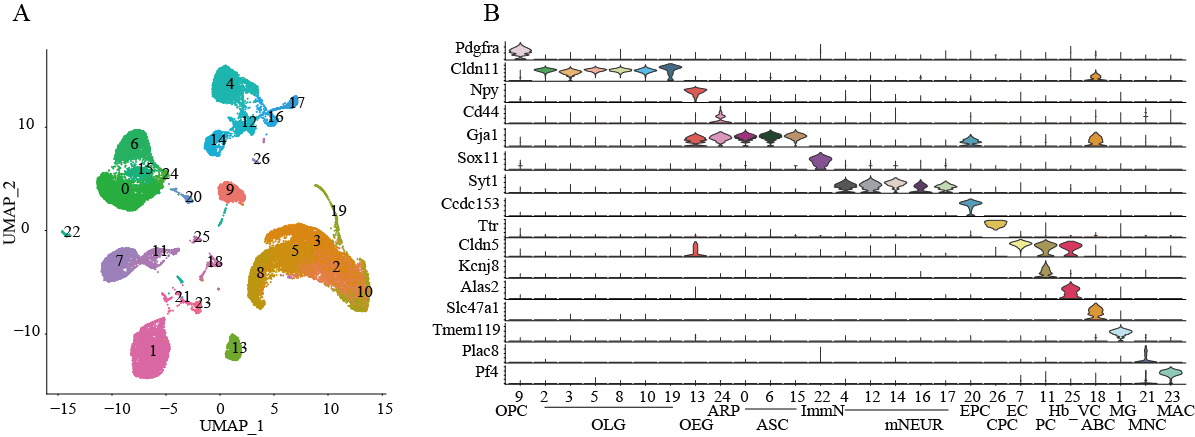


**Figure S6.** (A) UMAP plot of all cells, coloured by cluster numbers. (B) Violins showing expression of marker genes in different cell types.


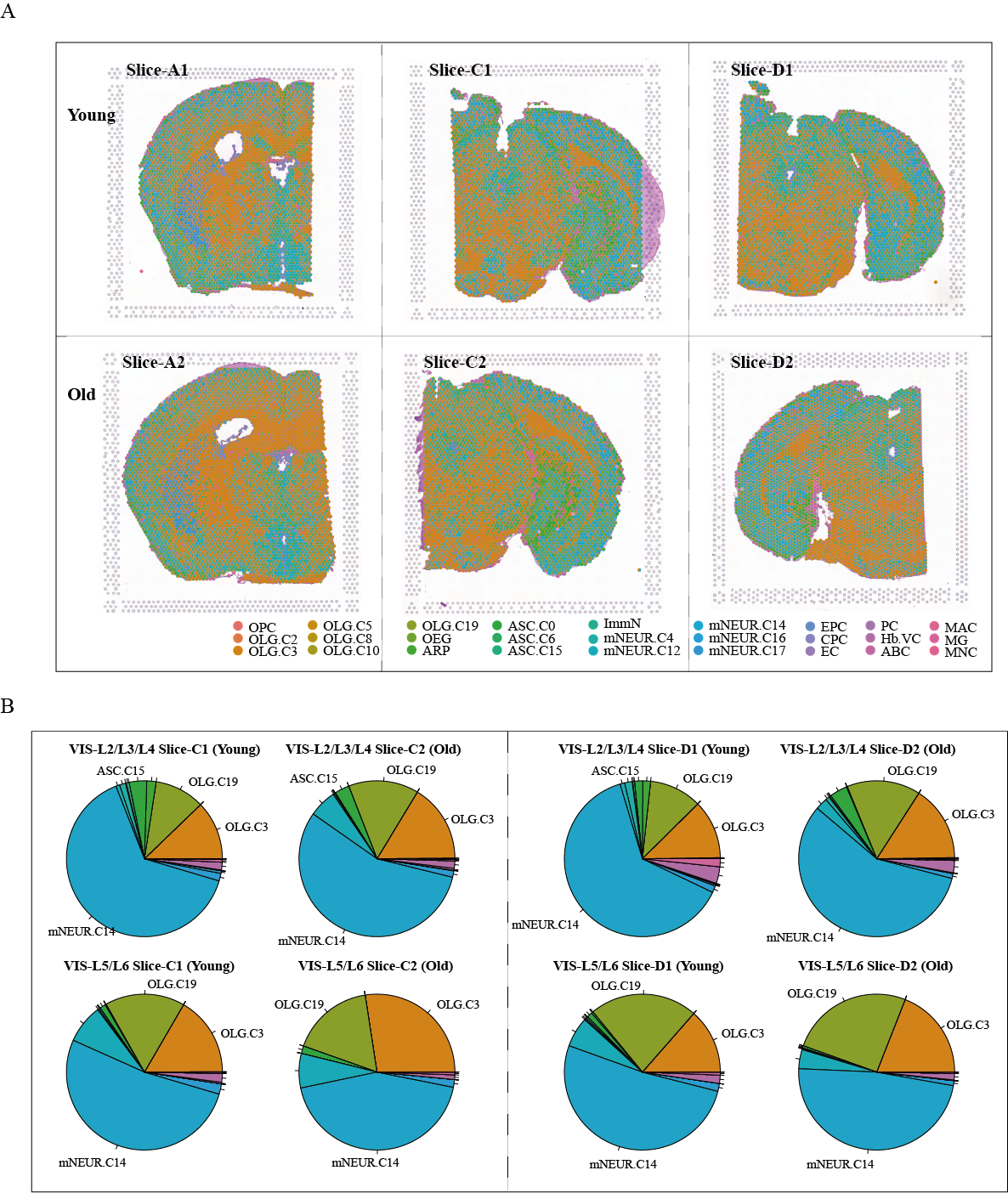


**Figure S7.** (A) Spatial distribution of cell types on different slices. (B) Pie chart showing the proportion of cell types contained in the VIS-L2/3/4 and VIS-L5/6 regions in other slices.


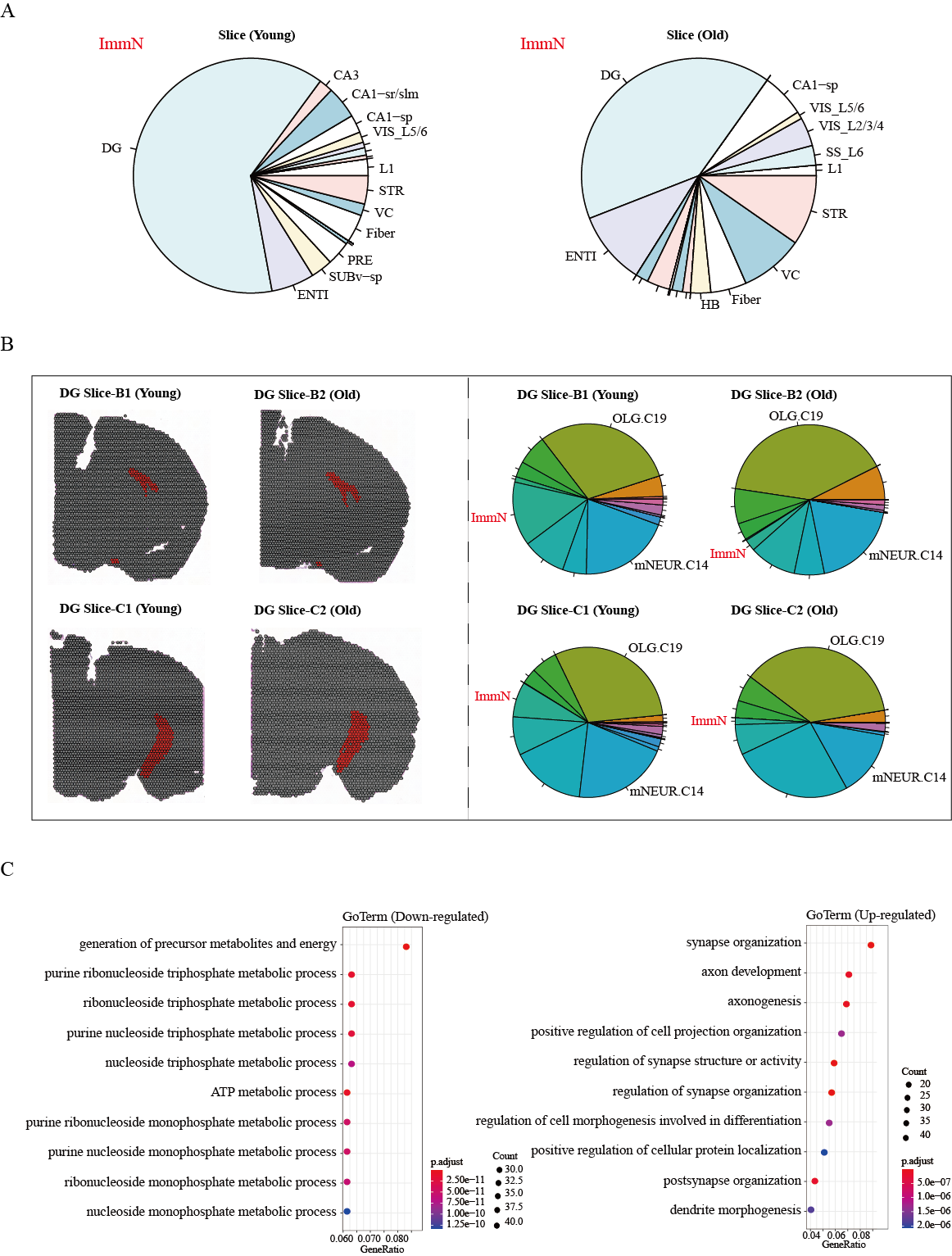


**Figure S8.** (A) Pie chart showing the proportion of ImmN in different regions, left (young sample), right (old sample). (B) Distribution of spots contained in the DG region on slice-B1, -B2, -C1 and -C2 (left), and the proportion of cell types in the DG region of ​​slice-B1, -B2, -C1 and -C2 (right). (C) Go enriched terms for up- and down-regulated genes in the DG region.


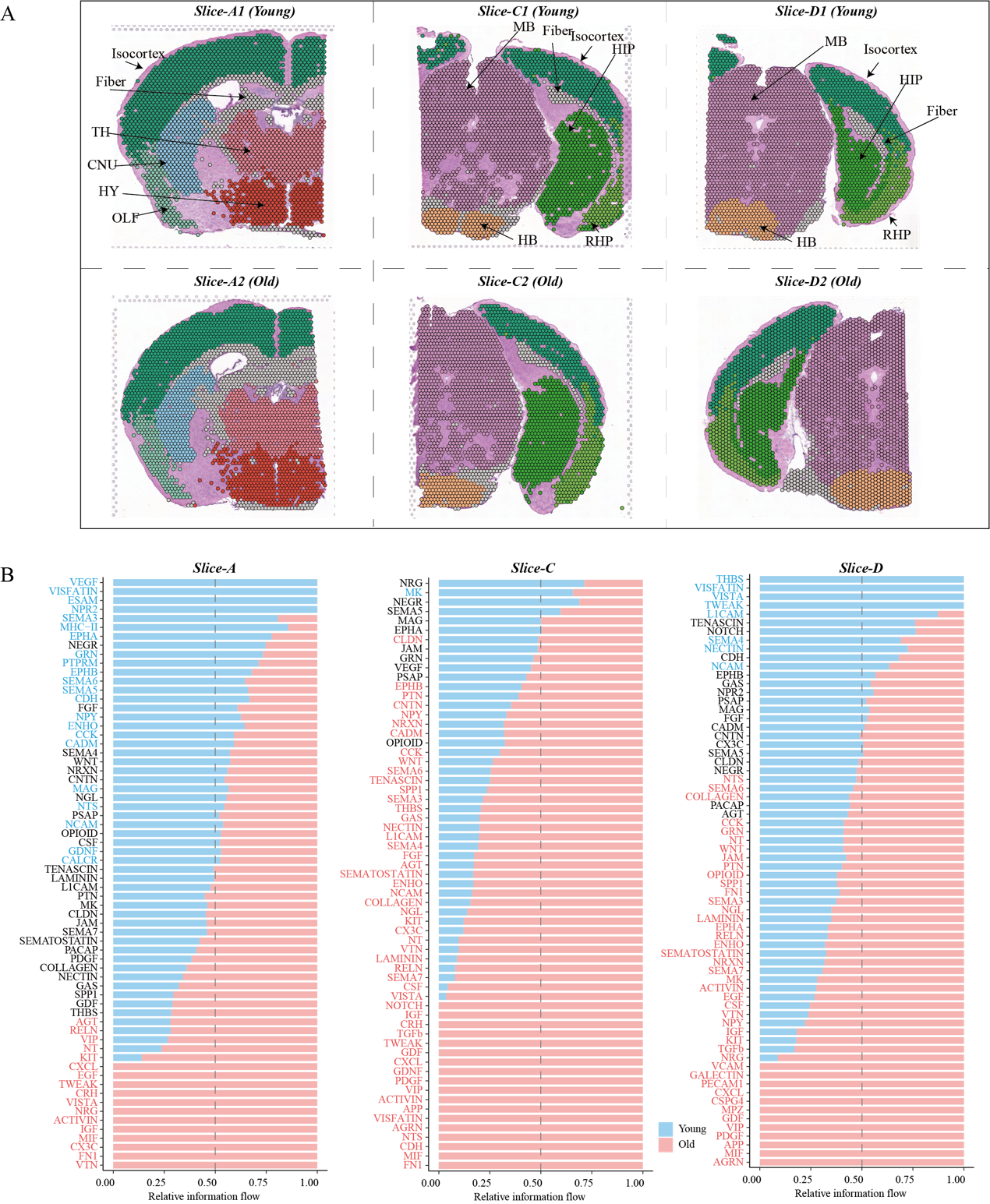


**Figure S9.** (A) Distribution of large regions on slices (slice-A1, -A2, -C1, -C2, D1 and D2). (B) All the significant signaling pathways were ranked based on their differences of overall information flow within the inferred networks between slice-A (left), slice-C (middle) and slice-D (right) from young and old mice. (C) Violin plot showing MIF signal-related ligand receptor gene expression in different regions.


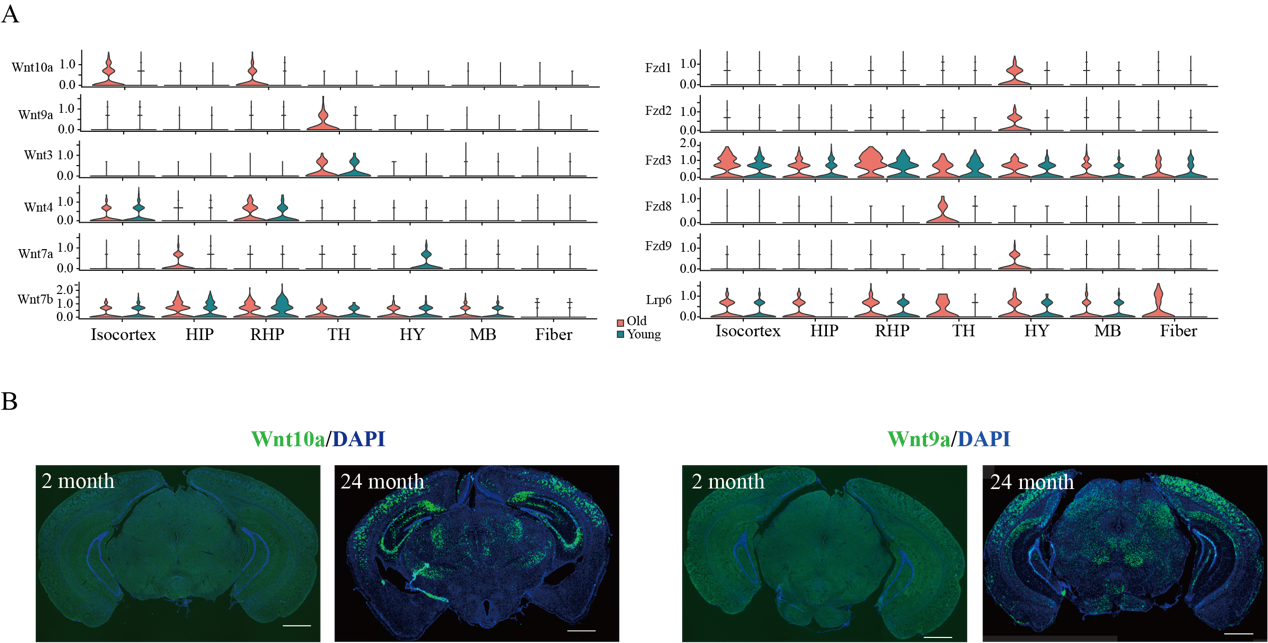


**Figure S10.** (A) Violin plot showing MIF signal-related ligand receptor gene expression in different regions. (B) Expression of the Wnt10 and Wnt9a in FFPE sections of young and old mice brains.


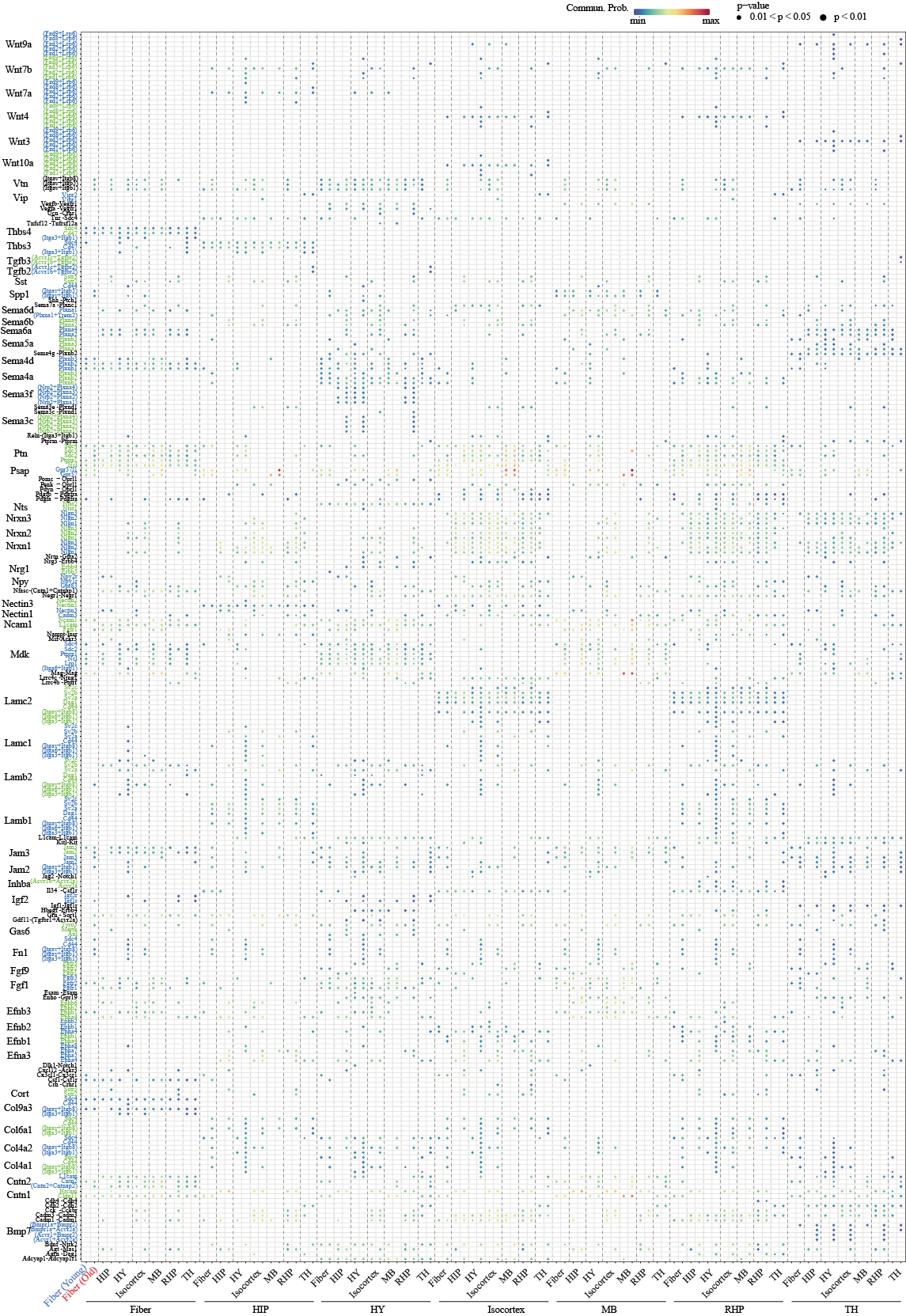


**Figure S11.** Comparison of the significant ligand-receptor pairs between different large regions in young and old mice.


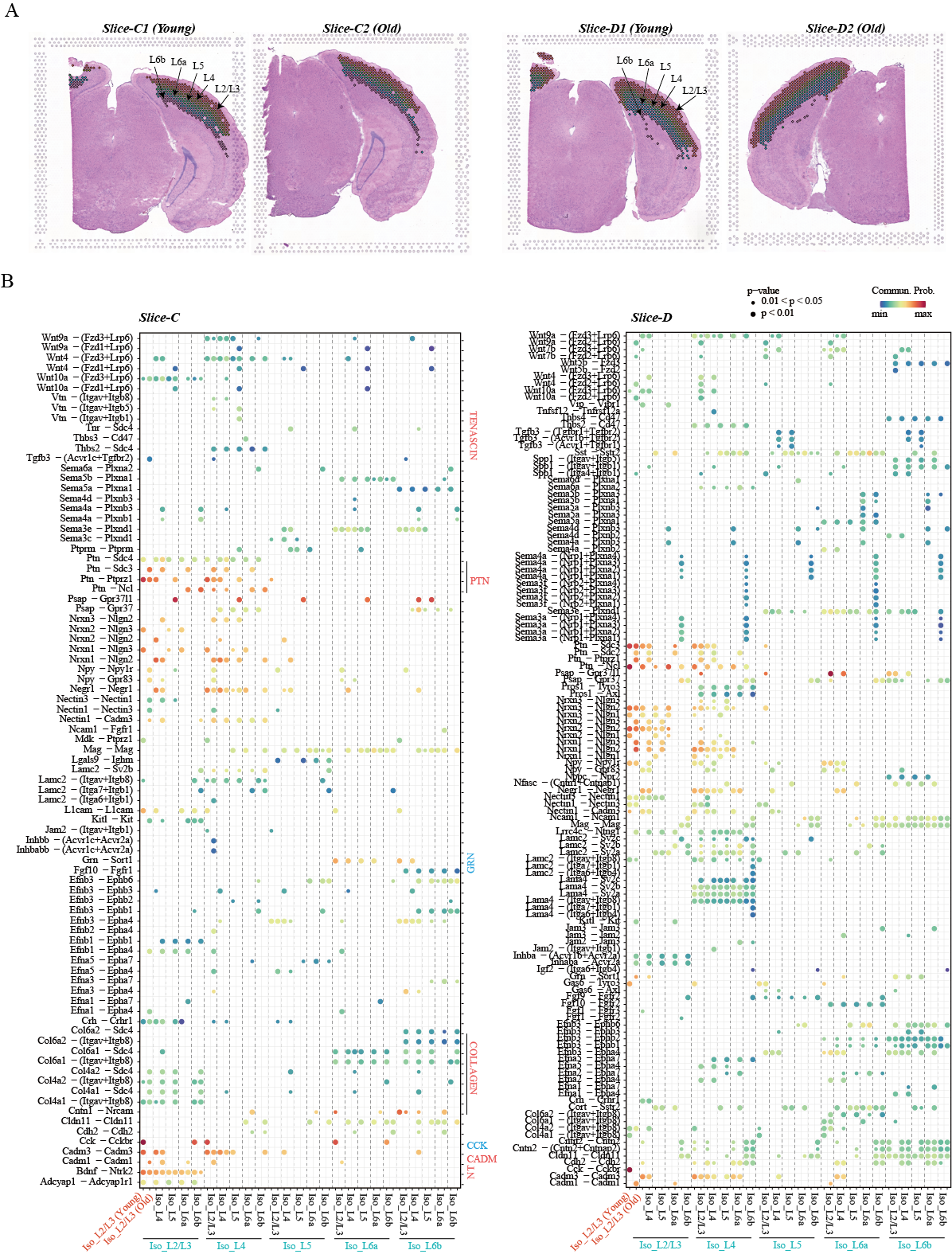


**Figure S12.** (A) Distribution of isocortex subregions on slices (slice-C1, -C2, -D1, -D2). (B) Comparison of the significant ligand-receptor pairs between slice-C (left) and slice-D (right) from young and old mice.


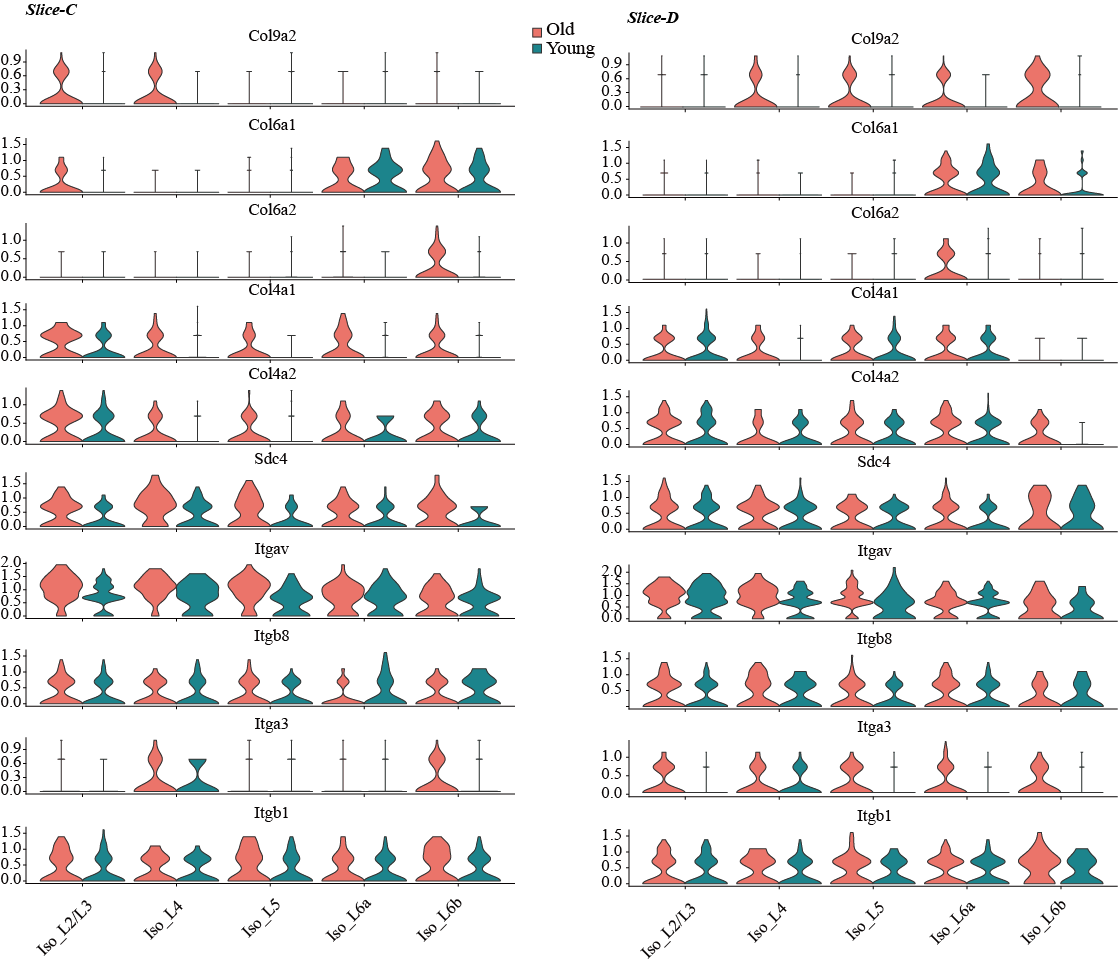


**Figure S13.** Violin plot showing COLLAGEN signal-related ligand receptor gene expression in different subregions from slice-C (left) and slice-D (right).

**Table S1**

The number of spots and genes contained in each slice.

| **Mouse** | **Sample** | **Coronal sections** | **Spots** | **Median reads per spot** | **Median genes per spot** |
| --- | --- | --- | --- | --- | --- |
| Young | Sample1 (left) | Slice-A1 | 3,259 | 113,172 | 5,360 |
|  | Sample2 (right) | Slice-B1 | 3,593 | 80,277 | 5,050 |
|  | Sample2 (right) | Slice-C1 | 3,400 | 108,233 | 4,744 |
|  | Sample2 (right) | Slice-D1 | 3,294 | 118,969 | 5,260 |
| Old | Sample3 (left) | Slice-A2 | 3,681 | 86,019 | 5,501 |
|  | Sample3 (right) | Slice-B2 | 3,543 | 97,791 | 5,655 |
|  | Sample3 (right) | Slice-C2 | 3,269 | 111,332 | 5,784 |
|  | Sample4 (left) | Slice-D2 | 3,050 | 102,922 | 6,028 |

**Table S2**

A set of genes involved in key signals associated with aging.

|  | **Gene sets** |
| --- | --- |
| Cellular senescence (GO:0090398) | Mtor, Eef1e1, Mapk14, Fzr1, Nuak1, Lmna, Icmt, Bcl6, Plk2, Prelp, Cdkn2aipnl, Prkcd, Cdkn1a, Calr, B2m, Ypel3, Map2k1, Mif, Hras, Id2, Zmiz1, Npm1, Terf2, Kras, Ybx1 |
| Positive regulation of inflammatory (GO:0050729) | Hspd1, Cd47, Fgfr1, Dhx9, Grn, Csf1r, Pde2a, Park7, Prkca, Ddt, Mdk, Il6st, Fcgr3, Lgals1, Fcer1g, Cebpb, Trem2, Nfkbia, Tac1, Il33, Cd81, Tnip1, Vamp8, Tnfrsf1a, Il18, Nupr1, Cdk19, Fem1a, Ldlr, Gprc5b, Cnr1, Rps19, Pdcd4, Kars, Snx4, App, Camk2n1, Snca, Ctss |
| Mitochondrial | mt-Co2, mt-Nd4, mt-Nd1, mt-Nd2, mt-Atp8, mt-Nd6, mt-Nd4l, mt-Nd5, mt-Nd3, mt−Co1, mt-Atp6, mt-Co3, mt-Cytb |
| Ribosomal | Rps26, Rps28, Rps29, Rpl9, Rpl37, Rpl10a, Rpl7l1, Rpl32, Rpl13a, Rpl7, Rpl31, Rpl12, Rps15, Rpl5, Rpl4, Rps20, Rpl21, Rps12, Rpl13, Rplp1, Rpl35, Rpl15, Rps15a, Rpl41, Rpl29, Rps2, Rpl36a, Rpl7a, Rpl27, Rpl19, Rps16, Rpl10, Rps27, Rpl18a, Rps25, Rps27l, Rpl22, Rpl23a, Rps6, Rpl34, Rpl24, Rps23, Rpl8, Rps11, Rpl27a, Rps4x, Rps17, Rps13, Rpl36al, Rps9, Rpl11, Rps14, Rpl18, Rpl36, Rpl6, Rpl14, Rplp2, Rpl28, Rps18, Rpl37a, Rpl17, Rpl26, Rpl23, Rpsa, Rplp0, Rpl3, Rpl22l1, Rps8, Rps5, Rps19, Rps10, Rps3, Rpl39, Rps7, Rps3a1, Rps27a, Rpl38, Rps21, Rpl35a, Rpl30, Rps24 |

**Table S3**

The oligonucleotide sequences used for qPCR.

| Primer Description | Primer sequence (5’-3’) |
| --- | --- |
| C1qa _F | AAAGGCAATCCAGGCAATATCA |
| C1qa _R | TGGTTCTGGTATGGACTCTCC |
| Cryab _F | GTTCTTCGGAGAGCACCTGTT |
| Cryab _R | GAGAGTCCGGTGTCAATCCAG |
| S100b _F | TGGTTGCCCTCATTGATGTCT |
| S100b _R | CCCATCCCCATCTTCGTCC |
| Bc1 _F | GTTGGGGATTTAGCTCAGTGG |
| Bc1 _R | AGGTTGTGTGTGCCAGTTACC |
| Tfrc _F | ATGCCGACAATAACATGAAGGC |
| Tfrc _R | ACACGCTTACAATAGCCCAGG |
| Eno1 _F | TGCGTCCACTGGCATCTAC |
| Eno1 _R | CAGAGCAGGCGCAATAGTTTTA |
| Penk _F | GGACTGCGCTAAATGCAGCTA |
| Penk _R | GAAGCCTCCGTACCGTTTCAT |
| Nrgn _F | TCCAAGCCAGACGACGATATT |
| Nrgn _R | CACACTCTCCGCTCTTTATCTTC |
| B2m _F | TTCTGGTGCTTGTCTCACTGA |
| B2m _R | CAGTATGTTCGGCTTCCCATTC |
| Ctss _F | CCATTGGGATCTCTGGAAGAAAA |
| Ctss _R | TCATGCCCACTTGGTAGGTAT |
| Ifi27 _F | TTCCCCCATTGGAGCCAAG |
| Ifi27 _R | AGGCTGCAATTCCTGAGGC |
